# Stromal Cell‐Mast Cell Communication Orchestrates Anti‐Viral Immunity in the Meninges

**DOI:** 10.1002/advs.202514842

**Published:** 2025-11-06

**Authors:** Qingqing Li, Weijia Chen, Mengxue Sun, Xinbo Ni, Qishan Ran, Xiaoyu Hu, Fang Cao, Wenwen Zeng

**Affiliations:** ^1^ Institute for Immunology and School of Basic Medical Sciences Beijing Key Laboratory of Immunological Research of Allergy (LIRA) Tsinghua University Beijing 100084 China; ^2^ Department of Neurosurgery Affiliated Hospital of Zunyi Medical University Zunyi 563000 China; ^3^ SXMU‐Tsinghua Collaborative Innovation Center for Frontier Medicine Shanxi Medical University Taiyuan 030001 China; ^4^ Tsinghua‐Peking Center for Life Sciences Beijing 100084 China

**Keywords:** IL‐33, mast cells, meninges, stromal cells, viral infection

## Abstract

Mast cells are tissue‐resident immune sentinels. However, their spatial localization and potential role in the antiviral response within the meninges—the protective barrier surrounding the central nervous system—remain unclear. Here, the distribution pattern along meningeal vasculature is maped and identified a post‐weaning maturation process. Single‐cell RNA sequencing reveals that mast cells mount a robust immune response against LCMV infection. Ablation of mast cells results in reduced CD8^+^ T cell infiltration and impairs viral clearance. Mechanistic dissection identifies a critical role for the IL‐33 receptor on mast cells, which responds to IL‐33 derived from stromal cells, in mediating antiviral immunity. Further analysis shows that mast cells synergistically upregulate cytokines and chemokines in response to IL‐33 and ATP released by virus‐infected stromal cells. Collectively, these findings reveal a critical role for mast cells in enhancing meningeal antiviral immunity and highlight potential strategies for brain protection during infection.

## Graphical abstract

This study delineates the spatial distribution and postnatal maturation of meningeal mast cells and uncovers their essential role in antiviral immunity. Using genetic models, mast cells are shown to promote CD8⁺ T cell infiltration and viral clearance via IL‐33 receptor signaling and synergistic activation by IL‐33 and ATP from infected stromal cells.

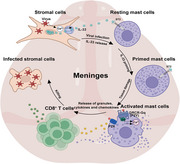

## 1 Introduction

Mast cells, best recognized for their dual roles in orchestrating type 2 immunity against helminth infections and driving pathogenic inflammation in allergic diseases, are strategically positioned at barrier sites such as the lung, skin, and gastrointestinal tract. Upon activation, these cells rapidly deploy pre‐stored mediators—including histamine, proteases, and cytokines—to regulate vascular permeability, leukocyte recruitment, and tissue remodeling, processes vital for host defense and allergic pathology.^[^ ^1^, ^2^, ^3^, ^4^, ^5^, ^6^ ^]^ Beyond classical barrier tissues, mast cells populate the dura mater of the meninges, a dynamic immunological interface that surveils the central nervous system (CNS).^[^ ^7^, ^8^ ^]^

Meningeal immunity is increasingly recognized as a hub for innate and adaptive immune cells.^[^ ^9^, ^10^, ^11^, ^12^, ^13^, ^14^, ^15^, ^16^, ^17^, ^18^, ^19^, ^20^, ^21^ ^]^ The functions of meningeal mast cells in various non‐infectious and bacterial infectious diseases have recently been revealed. In migraine, degranulation of meningeal mast cells contributes to the initiation and progression of disease by reinforcing a positive feedback loop of neurogenic inflammation with nociceptors.^[^ ^22^, ^23^, ^24^, ^25^ ^]^ In stroke, meningeal mast cells exacerbate stroke‐associated inflammation through degranulation, thereby promoting brain edema and immune cell infiltration.^[^ ^26^, ^27^, ^28^ ^]^ Notably, recent study has demonstrated that murine **Mrgprb2** and its human ortholog **MRGPRX2** sense the neuropeptide substance P, driving neutrophil migration into the brain parenchyma and worsening stroke outcomes.^[^ ^28^ ^]^ In bacterial meningitis, Mamuladze et al. have reported that degranulation of meningeal mast cells impairs cerebrospinal fluid (CSF) drainage after infection, thereby restricting bacterial dissemination through the arachnoid cuff exit (ACE) point into the parenchyma.^[^ ^29^ ^]^ However, the potential response of mast cells to viral infection at this critical CNS border remains unexplored.

Early evidence regarding the spectrum of chemokines and cytokines secreted by mast cells suggests their diverse roles in immunity.^[^ ^30^ ^]^ However, the precise nature of these roles is not fully understood. For instance, while mast cells are well known for mediating type 2 inflammation, which can impair antiviral responses,^[^ ^31^, ^32^, ^33^ ^]^ they can also be activated via receptors such as FcεRI (IgE‐dependent) and MRGPRX2 (IgE‐independent) to respond to pathogens, neuropeptides, and danger signals like ATP.^[^ ^34^, ^35^, ^36^, ^37^, ^38^ ^]^ Nevertheless, the molecular drivers of mast cell responses during viral infection and their functional heterogeneity across tissues remain unclear^[^ ^3^, ^39^, ^40^, ^41^, ^42^ ^]^—representing a critical gap in our understanding of the meninges, where such intercellular networks are crucial for shaping CNS immunity.

Here, we define meningeal mast cells as crucial regulators of antiviral defense during lymphocytic choriomeningitis virus (LCMV) Armstrong infection. Spatial and single‐cell transcriptomic analyses revealed their maturation pattern during development and activation signatures post‐infection. Contrary to the paradigm that type 2‐associated cells impede antiviral immunity, specific mast cell depletion impaired viral clearance and CD8^+^ T recruitment to the meninges, whereas chemogenetic or optogenetic activation augmented CD8^+^ T cell infiltration. By developing a *Tpsb2‐CreERT2* mouse line for mast cell‐specific perturbation, we identified IL‐33/ST2 signaling as a crucial mechanism, whereby stromal cell‐derived IL‐33 licensed mast cells to amplify chemokine and cytokine production in response to ATP. Our findings establish mast cells as orchestrators of CNS border immunity, unveiling a stromal‐immune axis important for anti‐viral immune response, and highlight therapeutic targets for meningeal inflammation.

## 2 Results

### 2.1 Tpsb2‐CreERT2 Enables Efficient and Specific Labeling of Mast Cells, Revealing their Meningeal Distribution

**Figure 1:**
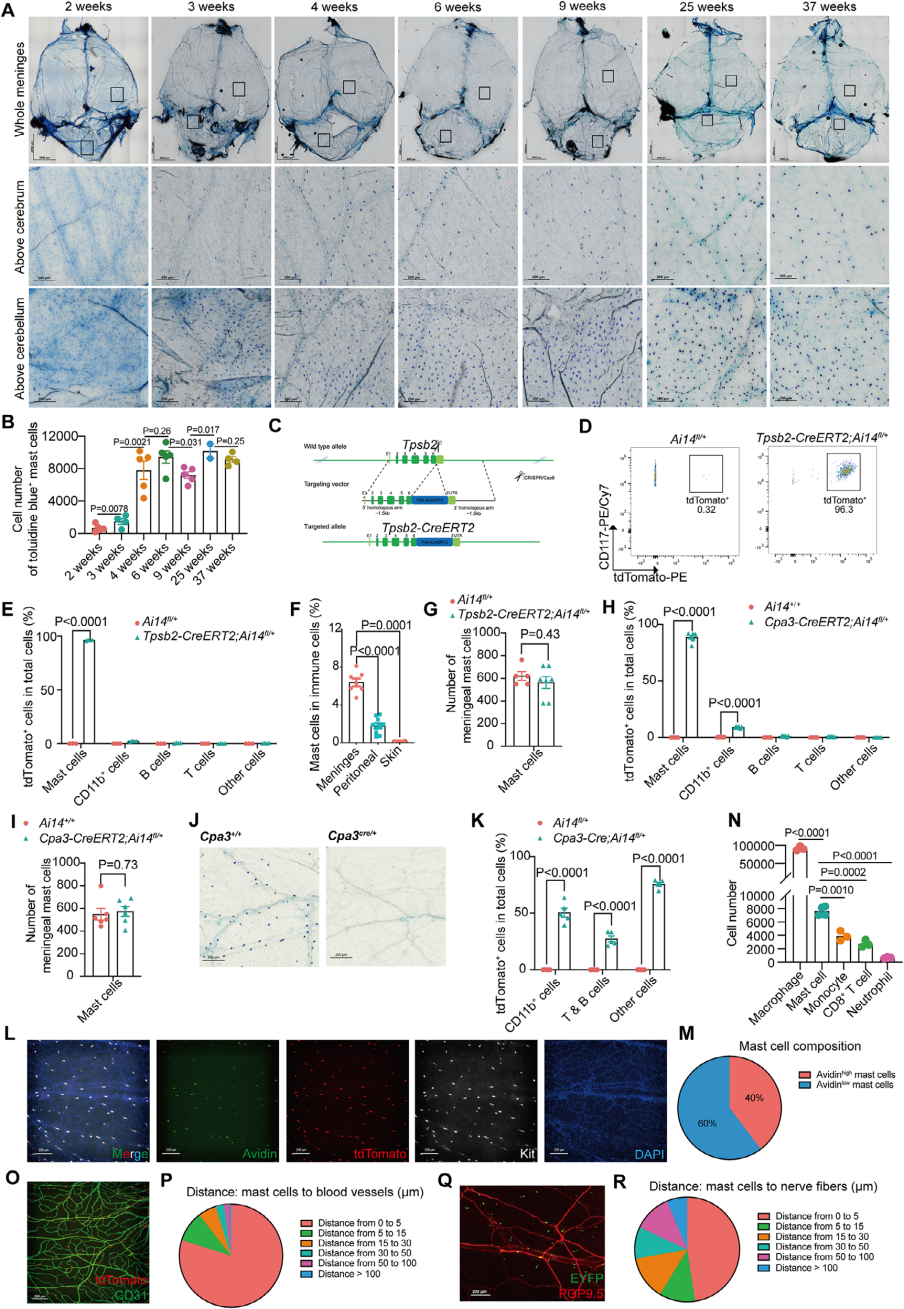
*Tpsb2‐CreERT2* enables efficient and specific labeling of mast cells, revealing their meningeal distribution. A) Minimal intensity projection of dural meninges stained with toluidine blue from mice at different developmental stages. n = 2–5 (mice). B) Quantification of (A). C) Targeting strategy of *Tpsb2‐CreERT2* mice. D) Representative contour plot of meningeal CD45+CD11b‐CD3e‐CD19‐ cells from *Tpsb2‐CreERT2; Ai14^fl/+^* mice. n = 3–4 (mice). E) Quantification of labeled immune cell subset in the meninges. n = 3–4 (mice). F) The percentage of mast cells in different tissues. n = 2–11 (mice). G) The number of meningeal mast cells from *Tpsb2‐CreERT2; Ai14^fl/+^* mice. n = 5–7 (mice). H) Quantification of labeled immune cell subset in the meninges from *Cpa3‐CreERT2; Ai14^fl/+^* mice. n = 6 (mice). I) The number of meningeal mast cells from *Cpa3‐CreERT2; Ai14^fl/+^* mice. n = 6 (mice). J) Minimal intensity projection of dural meninges stained with toluidine blue from *Cpa3^cre/+^* mice. n = 3 (mice). K) Quantification of labeled immune cell subset in the meninges from *Cpa3‐Cre; Ai14^fl/+^* mice. n = 4–6 (mice). L) Maximal intensity projection of dural meninges stained for granule (green), mast cell (red, white), and nuclei (blue) from *Tpsb2‐CreERT2; Ai14^fl/+^* mice. n = 6 (mice). M) Quantification of (L). N) Quantification of macrophages, mast cells, monocytes, CD8⁺ T cells, and neutrophils in the dural meninges. n = 3–4 (mice). O) Maximal intensity projection of dural meninges stained for mast cells (red) and blood vessels (green) from *Tpsb2‐CreERT2; Ai14^fl/+^* mice. n = 3 (mice). P) Quantification of (O). Q) Spatial distribution of mast cells (green) and nerve fibers (red) in meninges from *Tpsb2‐CreERT2; Ai32^fl/fl^* mice. n = 3 (mice). R) Quantification of (Q). Scale bar for all images, 2000 µm (whole mount), 200 µm (partial region). Data are presented as mean ± SEM and *p* values were calculated by two‐tailed, unpaired Student's t‐test (B, E–I, K, N).

To delineate meningeal mast cell functions, we first validated their presence in mice. Toluidine blue revealed a gradual increase in meningeal mast cell numbers during postnatal development from 2 to 4 weeks of age, stabilizing at elevated levels after 6 weeks (**Figure** **1**A,B).

We next assessed meningeal mast cell‐specific transcriptional signatures using published sequencing datasets.^[^ ^43^, ^44^, ^45^ ^]^ These cells exhibited high expression of tryptase β2 (*Tpsb2*), tryptase α/β1 (*Tpsab1*), chymase 1 (*Cma1*), and mast cell protease 4 (*Mcpt4*), consistent with connective tissue mast cell characteristics (Figure S1A, Supporting Information). In contrast, they showed minimal expression of protease 1 (*Mcpt1*) or protease 2 (*Mcpt2*), the hallmark genes of mucosal mast cells, suggesting distinct regulatory mechanisms compared to mucosal immunity.

To target meningeal mast cells, we generated *Tpsb2‐CreERT2* mice by inserting a CreERT2 expression cassette downstream of *Tpsb2* locus (Figure 1C). When crossed with *Ai14* reporter mice, FACS analysis of meninges, peritoneal cavity and skin showed 96% tdTomato labeling efficiency in total mast cells (CD117^+^FcεRIα^+^) (Figure 1D,E; Figure S1B–D, Supporting Information). Meningeal mast cells constituted ≈7% of total CD45^+^ immune cells under steady‐state conditions—significantly higher than in the peritoneal cavity (≈2%) or skin (≈0.2%) (Figure 1F).

Comparable mast cell numbers in wild‐type and *Tpsb2‐CreERT2; Ai14* meninges suggested no apparent Cre‐driven cytotoxicity (Figure 1G). Transcriptomic profiling of peritoneal mast cells further confirmed no significant changes in proliferation, growth, differentiation or cell death pathways (Figure S1E, Supporting Information). Thus, the *Tpsb2‐CreERT2* system did not alter mast cell viability or development.

For comparison, *Cpa3‐creERT2* and *Cpa3‐cre* lines crossed with *Ai14* mice revealed that *Cpa3‐creERT2* labeled mast cells in the meninges and peritoneal cavity but exhibited non‐specific expression in a subset of meningeal CD11b^+^ myeloid cells (Figure 1H,I; Figure S1F, Supporting Information). In contrast, the *Cpa3‐cre* line significantly reduced mast cell numbers and non‐specifically labeled granulocytes and lymphocytes (Figure 1J,K; Figure S1G,H, Supporting Information).

TdTomato staining in *Tpsb2‐CreERT2; Ai14* mice co‐localized strongly with Kit, confirming targeting specificity (Figure 1L). Notably, avidin labeling—widely used to identify mast cells—can easily underestimate meningeal mast cell numbers due to heterogeneity. While genetic labeling using *Tpsb2‐CreERT2; Ai14* identified all mast cells, only 40% showed strong avidin labeling; the remaining 60% exhibited a weak avidin signal, though all cells stained positive to some degree (Figure 1L,M). Since avidin binds strongly to granule content,^[^ ^46^, ^47^ ^]^ avidin^high^ cells likely represent highly granulated populations.

Whole‐mount immunofluorescence staining quantified meningeal mast cells (7640 ± 349) as significantly more abundant than Ly6C^+^ monocytes (3893 ± 421), CD8⁺ T cells (2753 ± 314), and Ly6G^+^ neutrophils (679 ± 76), and were only surpassed by CD206^+^ macrophages (91301 ± 3436) among the assessed immune cell subtypes (Figure 1N; Figure S1I–L, Supporting Information). These results indicate that prior scRNA‐seq studies may underestimate mast cells abundance due to isolation limitations.^[^ ^43^ ^]^

Positional analysis indicated that meningeal mast cells localized near macrophages (average 6.28 ± 0.12 µm) but were distant from monocytes (average 111.55 ± 0.59 µm), CD8⁺ T cells (average 147.65 ± 0.95 µm), and neutrophils (average 416.83 ± 1.69 µm) (Figure S1M–P, Supporting Information). They also resided near perivascular sites and vascular branch points (average 5.26 ± 0.13 µm), implying roles in vascular permeability and immune cell infiltration (Figure 1O,P). Human meningeal mast cells similarly localized near CD31^+^ vessels and expressed tryptase (Figure S1Q, Supporting Information).

Using *Tpsb2‐CreERT2; Ai32* mice, mast cells were detected within 24.06 ± 1.72 µm of nerve fibers immunostained for ubiquitin carboxyl‐terminal hydrolase‐1 (UCH‐L1, also known as PGP9.5) (Figure 1Q,R). Though functional implications were unexplored, this proximity suggests interactions with neuronal structure in neuroimmune regulation.

In summary, we elucidated spatiotemporal distribution of meningeal mast cells and provided a foundation for functional interrogation.

### 2.2 Meningeal Mast Cells Mount a Potent Immune Response to Viral Infection

The meninges serve as a protective barrier for the CNS and are essential in combating pathogen attacks, such as viral infections, which can otherwise cause fatal infections. Recent studies have unveiled meningeal immune responses that exhibit distinct features compared to those in the CNS parenchyma or peripheral organs.^[^ ^9^, ^20^ ^]^ To investigate these responses during viral infections, we employed an acute model using LCMV Armstrong.

Following intravenous LCMV inoculation in adult mice, meningeal viral load gradually increased from day 0 to day 6, as measured by quantitative PCR (qPCR), and was cleared by 6 days post‐infection (dpi) (Figure S2A, Supporting Information). Concurrently, CD8⁺ T cell and monocyte numbers in the meninges peaked at 6 dpi (Figure S2B,H, Supporting Information). In contrast, the virus was rapidly cleared from the spleen within 2 days (Figure S2C, Supporting Information). The spleen and peripheral blood developed immune responses marked by increased CD8⁺ T cells, monocytes, and neutrophils, alongside reduced splenic viral load (Figure S2C–G,I–J, Supporting Information).

Whole‐mount staining revealed scattered mast cells and macrophages throughout the meninges, with a slight numerical reduction, possibly due to virus‐induced cell death or migration (Figure S3A, Supporting Information).^[^ ^18^, ^19^ ^]^ Recruited monocytes, CD8⁺ T cells, and neutrophils, however, exhibited clustered distribution (Figure S3B–D, Supporting Information), suggesting that resident cells remain dispersed while newly recruited immune cells aggregate to mount effective antiviral immunity.

**Figure 2:**
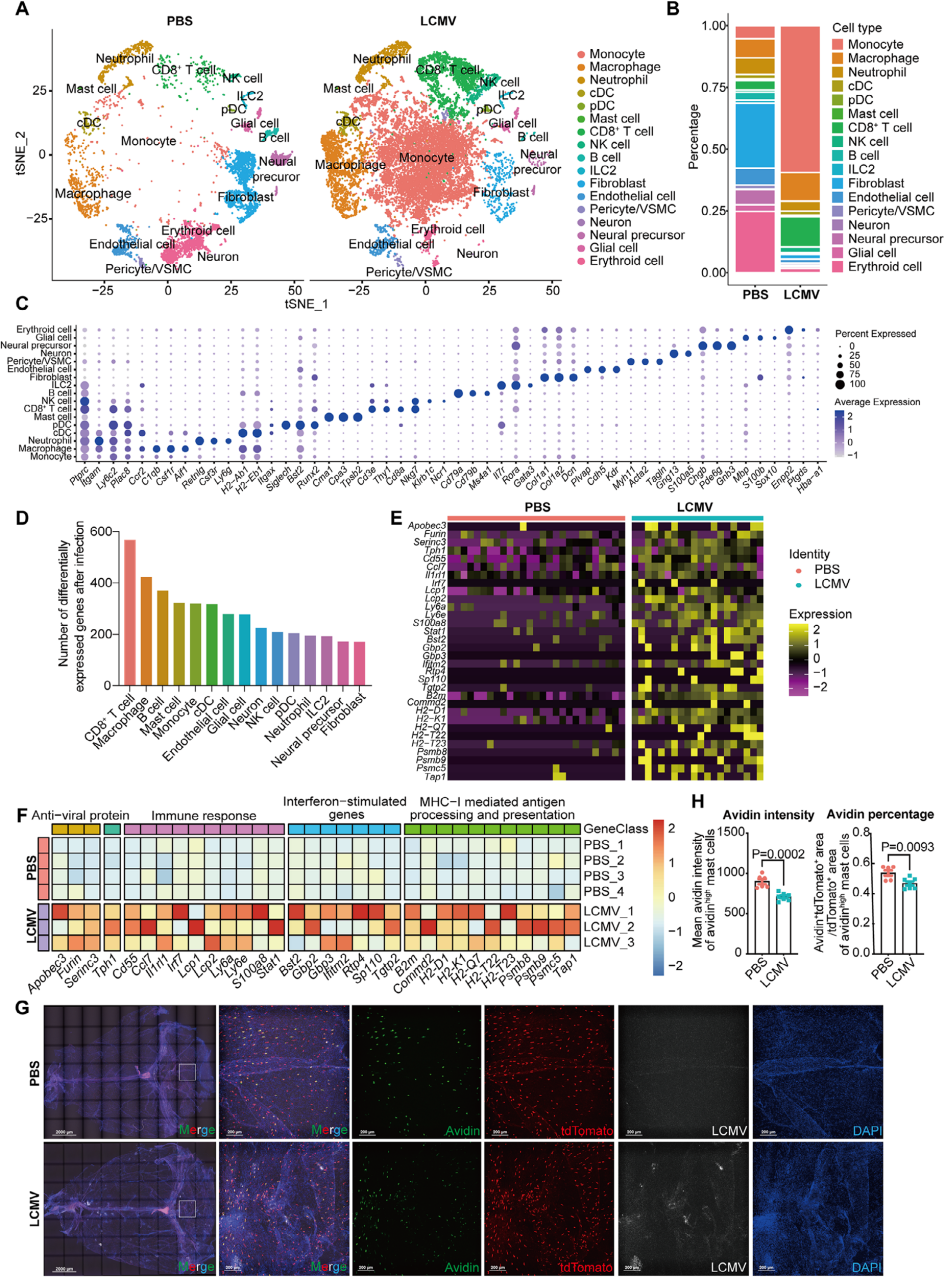
Meningeal mast cells mount a potent immune response to viral infection. A) t‐Distributed stochastic neighbor embedding (t‐SNE) visualization of sc‐RNA seq of meningeal cells from mice un‐infected (6623 cells) or infected (20857 cells) with LCMV at 6 dpi. B) Percentage of indicated cell clusters in the total cells from mice un‐infected or infected with LCMV. C) Marker genes dot plot of indicated cell clusters. D) The number of differentially expressed genes in various cell types from mice un‐infected or infected with LCMV. E) Heatmap of differentially expressed genes in mast cells analyzed by sc‐RNA seq from mice un‐infected or infected with LCMV. F) Heatmap of differentially expressed genes in mast cells analyzed by SMART‐seq from mice un‐infected or infected with LCMV. n = 3–4 (mice). G) Maximal intensity projection of dural meninges stained for granules (green), mast cells (red), LCMV (white) and nuclei (blue) from *Tpsb2‐CreERT2; Ai14^fl/+^* mice un‐infected or infected with LCMV at 6 dpi. Scale bar, 2000 µm (left, whole mount); 200 µm (right). n = 6–7 (mice). H) Average granulation extent of dural mast cells analyzed by mean intensity or area of avidin^high^ granules (G). Data are presented as mean ± SEM and *p* values were calculated by Mann‐Whitney U test from Seurat (D, E), Wald test from DESeq2 (F), two‐tailed, unpaired Student's *t*‐test (H).

To further characterize meningeal immune response during peak activity (6 dpi), we performed scRNA‐seq. Increased proportions of monocytes and CD8⁺ T cells, along with enhanced cell‐cell communications, indicated meningitis induction (**Figure** **2**A–C; Figure S3E,F, Supporting Information). Notably, signaling interactions from mast cells to CD8⁺ T cells were strengthened after infection, suggesting a potential regulatory role of mast cells in CD8⁺ T cell responses (Figure S3F, Supporting Information). Differentially expressed gene analysis across cell types revealed upregulation in CD8⁺ T cells, macrophages, B cells, mast cells, and monocytes (Figure 2D). Mast cells—in addition to macrophages, B cells, and monocytes—showed elevated expression of antiviral proteins, immune mediators, interferon‐stimulated genes (ISGs), and antigen‐processing and ‐presentation proteins (Figure 2E), indicating their robust immune engagement and critical role in antiviral immunity.

We validated these findings using SMART‐seq (RNA‐seq) of meningeal mast cells sorted at 6 dpi. Transcriptomic profiles confirmed scRNA‐seq results (Figure 2F; Figure S3G, Supporting Information), with specific upregulation of *Furin*, *Tph1* (tryptophan hydroxylase 1), *Cd55*, *Ccl7*, and *Il1rl1* in mast cells.

Notably, granule contents labeled by avidin were significantly reduced, consistent with active degranulation by 6 dpi (Figure 2G,H; Figure S3H–K, Supporting Information). Whole‐mount staining also revealed a slight decrease in tdTomato^+^ meningeal mast cells post‐infection, potentially reflecting partial cell death or migration during the antiviral response (Figure 2G; Figure S3H, Supporting Information).

Collectively, we show that meningeal mast cells undergo profound transcriptional reprogramming and degranulation in response to LCMV infection, mounting a potent and multifaceted antiviral immune response.

### 2.3 Mast Cell Activation Promotes Immune Cell Recruitment to The Meninges

**Figure 3:**
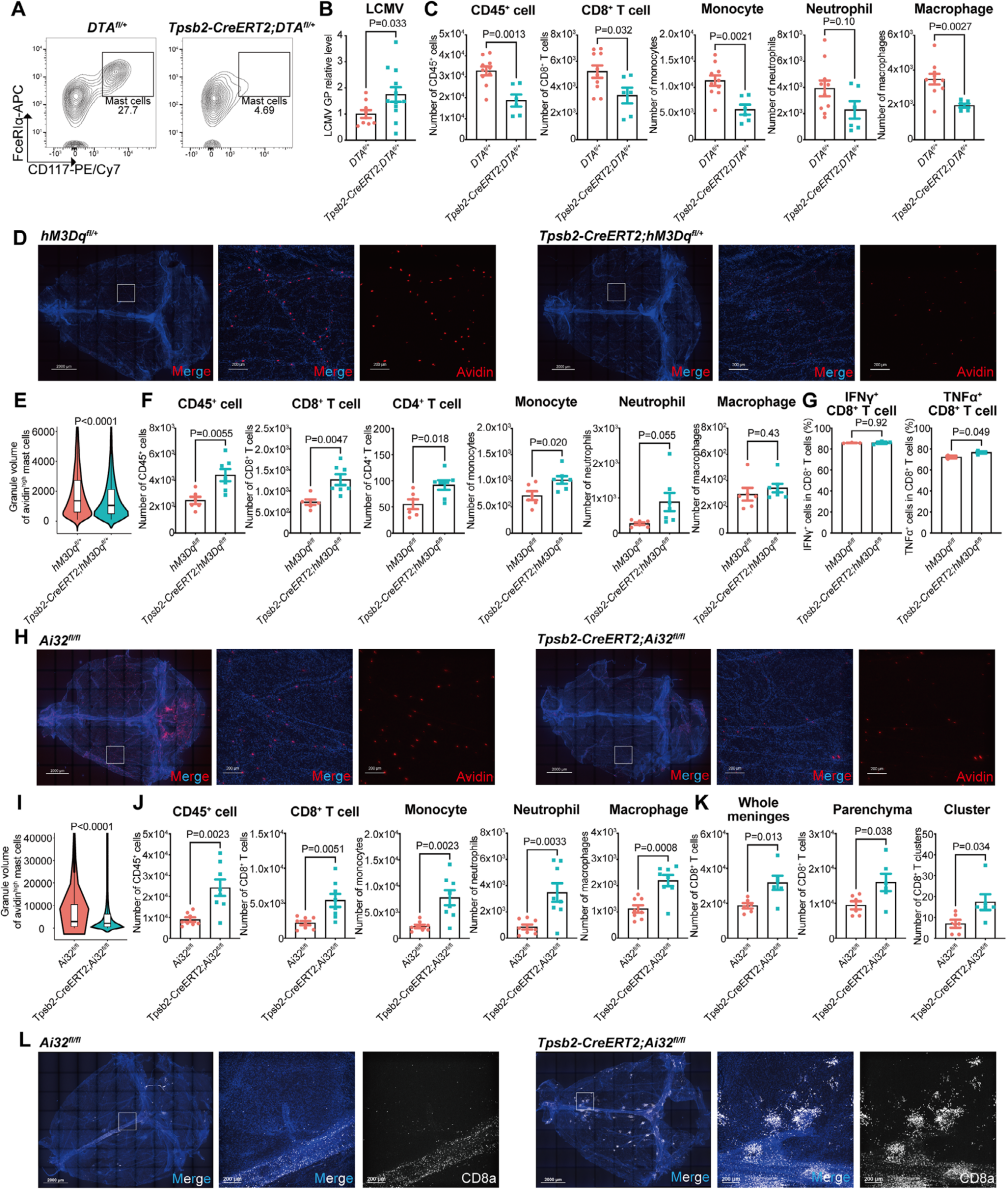
Mast cell activation promotes immune cell recruitment to the meninges. A–C) Depletion of mast cells was employed with *Tpsb2‐CreERT2; DTA^fl/+^* mice. A) Representative contour plot of meningeal CD45^+^CD11b^−^CD3e^−^CD19^−^ cells. n = 6 (mice). B) LCMV mRNA level in the dural meninges at 6 dpi. n = 9–12 (mice). C) Meningeal cell counts at 6 dpi. n = 6–10 (mice). D–G) Pharmacogenetic activation of mast cells was employed with *Tpsb2‐CreERT2; hM3Dq^fl/+^* mice. D) Maximal intensity projection of dural meninges stained for granule (red) and nuclei (blue) at 5.6 dpi. n = 4–6 (mice). E) Granulated volume of avidin^high^ mast cells analyzed by avidin^+^ volume (D). n = 6334‐6081 (cells). F) Meningeal cell counts at 5.6 dpi. n = 6–7 (mice). G) The IFN‐γ and TNF‐α production by meningeal CD8^+^ T cells at 5.6 dpi. n = 3 (one point from 2 mice). H–L) Optogenetic activation of mast cells was employed with *Tpsb2‐CreERT2; Ai32^fl/fl^* mice. H) Maximal intensity projection of dural meninges stained for granule (red) and nuclei (blue) at 5.6 dpi. n = 3–6 (mice). I) Granulated volume of avidin^high^ mast cells analyzed by avidin^+^ volume (H). n = 12442‐22514 (cells). J) Meningeal cell counts at 5.6 dpi. n = 8 (mice). K) The number of CD8^+^ T cells (L). L) Maximal intensity projection of dural meninges stained for CD8^+^ T cells (white) and nuclei (blue) at 5.6 dpi. n = 6 (mice). Scale bar for all images, 2000 µm (whole mount), 200 µm (partial region). Data are presented as mean ± SEM and *p* values were calculated by two‐tailed, unpaired Student's *t*‐test (B,C, E–G, I–K).

To investigate the role of mast cells in antiviral immunity, we generated *Tpsb2‐CreERT2; Rosa26‐DTA* mice, in which mast cells were selectively ablated following tamoxifen treatment (**Figure** **3**A; Figure S4A, Supporting Information). Upon viral infection, impaired antiviral responses in the meninges—but not in the spleen or blood—were confirmed by reduced immune cell infiltration and elevated viral titers, consistent with an important role of mast cells in meningeal antiviral immunity (Figure 3B,C; Figure S4B–F, Supporting Information). Collectively, these data underscore the importance of meningeal mast cells and highlight the advantage of targeted genetic deletion for specific perturbation.

Next, we recapitulated mast cell activation by expressing hM3Dq (DREADD engineering Gq) in meningeal mast cells (Figure S5A–C, Supporting Information). Following viral infection, Gq pathway activation via clozapine N‐oxide (CNO) administration enhanced the meningeal antiviral immune response, evidenced by increased recruitment of CD8⁺ T cells, CD4⁺ T cells, and monocytes to the meninges (Figure 3D–G; Figure S5D–I, Supporting Information).

Furthermore, we developed an optogenetic approach using ChR2 to achieve localized, specific activation of meningeal mast cells in vivo. In *Tpsb2‐CreERT2; Ai32* mice but not wild‐type controls, LED light stimulation (470 nm) of the meninges enhanced mast cell degranulation in vivo and in peritoneal cell‐derived mast cells (PCMCs) in vitro (Figure S5J–N, Supporting Information). During viral infection, optogenetic activation significantly amplified degranulation and triggered robust immune cell, including CD8⁺ T cells, monocytes, neutrophils, and macrophages into the meninges (Figure 3H–J; Figure S5O, Supporting Information). In contrast, no significant changes in immune cell composition occurred in the spleen or peripheral blood (Figure S5P–R, Supporting Information). Whole‐mount immunostaining revealed a 2‐fold increase in CD8⁺ T cell infiltration into the meningeal parenchyma. Notably, CD8⁺ T cells formed cellular clusters comprising (>50 cells) continuous aggregates in the infected meninges, a phenomenon further augmented by mast cell activation (Figure 3K,L).

Together, these complementary genetic and optogenetic models provided evidence that meningeal mast cells critically mediate immune cell recruitment and coordinate effective antiviral immune responses.

### 2.4 ST2 Signal on Meningeal Mast Cells Promotes the Antiviral Immune Response and CD8^+^ T Cell Infiltration

Next, we sought to unravel the molecular mechanism underlying mast cell activation and its role in regulating antiviral immunity. Mast cells can be activated by surface receptors, leading to cytokine and chemokine production and degranulation.^[^ ^48^ ^]^ ST2 (encoded by *Il1rl1*), an IL‐33 receptor subunit, is well‐characterized in group 2 innate lymphoid cells (ILC2s) and T cells.^[^ ^49^, ^50^ ^]^ Notably, we found ST2 highly expressed in meningeal mast cells and ILC2s, with mast cell expression upregulated after infection (Figure S6A, Supporting Information). Similarly, ST2 was specifically and highly expressed in human meningeal mast cells, suggesting a conserved role in the meninges (Figure S6B, Supporting Information).

**Figure 4:**
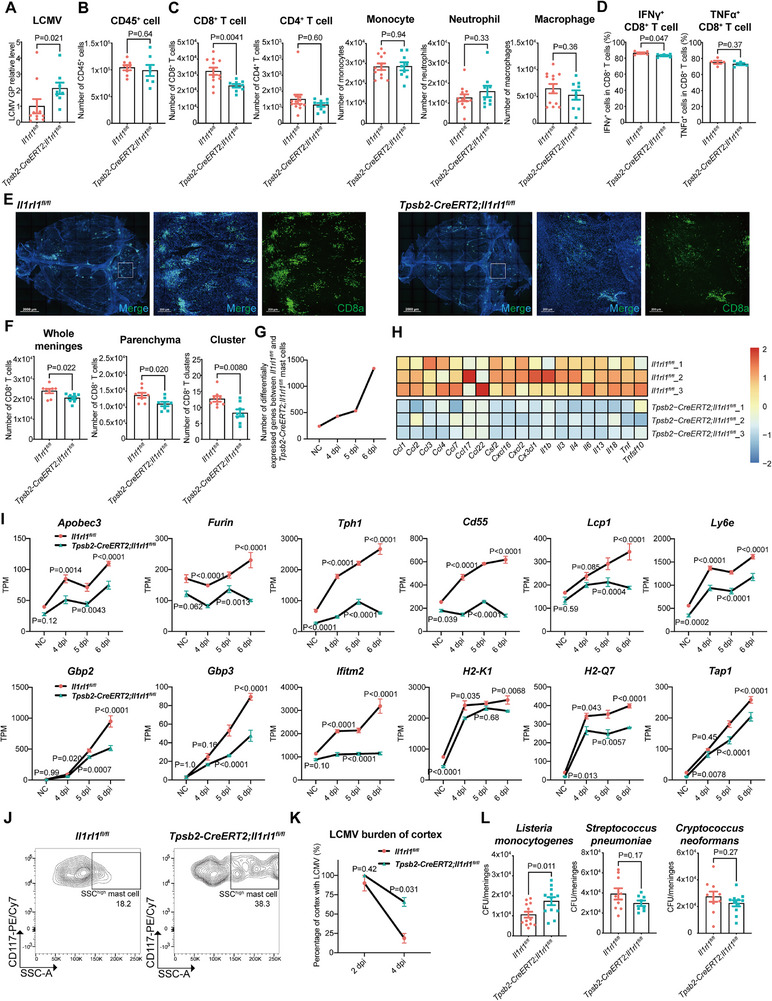
ST2 signal on meningeal mast cells promotes the antiviral immune response and CD8^+^ T cell infiltration. A–L) *Tpsb2‐CreERT2; Il1rl1^fl/fl^* mice were used to delete ST2 in mast cell. A) LCMV mRNA level in the dural meninges at 6 dpi. n = 8–10 (mice). B) Meningeal immune cell counts using whole mount CD45 staining at 6 dpi. n = 7–8 (mice). C) Meningeal cell calculated counts using CD45 staining and FACS at 6 dpi. n = 9–12 (mice). D) The IFN‐γ and TNF‐α production by meningeal CD8⁺ T cells at 6 dpi. n = 5 (one point from 2 mice). E) Maximal intensity projection of dural meninges stained for CD8⁺ T cells (green) and nuclei (blue) at 6 dpi. Scale bar, 2000 µm (left, whole mount); 200 µm (right). n = 8 (mice). F) The number of CD8⁺ T cells (E). G) The number of differentially expressed genes in meningeal mast cells at 0, 4, 5, 6 dpi. n = 3 (mice). H) Heatmap of differentially expressed cytokines and chemokines in meningeal mast cells at 6 dpi. n = 3 (mice). I) TPM values of differentially expressed genes in meningeal mast cells at 0, 4, 5, 6 dpi. n = 3 (mice). J) Representative contour plot of meningeal mast cells at 6 dpi. n = 3 (mice). K) LCMV load in the cortex at 2 and 4 days post second viral challenge. n = 2 (experiments, each experiment from 4–8 mice). L) *Listeria monocytogenes*, *Streptococcus pneumoniae*, *Cryptococcus neoformans* load in the dural meninges at 48 hours post infection. n = 8–13 (mice). Data are presented as mean ± SEM and *p* values were calculated by two‐tailed, unpaired Student's t‐test (A–D, F, K,L), Wald test from DESeq2 (G–I).

To investigate the functional significance of ST2, we generated mice with mast cell‐specific *Il1rl1* ablation (*Tpsb2‐CreERT2; Il1rl1^fl/fl^*) and validated knockout efficiency (Figure S6C, Supporting Information). Following LCMV infections, ST2 deletion in mast cells resulted in higher meningeal viral loads and reduced CD8⁺ T cell infiltration assessed by RT‐qPCR, staining and FACS, with no major differences in other cell types (**Figure** **4**A–C; Figure S6D, Supporting Information). Furthermore, ST2 deficiency impaired interferon γ (IFN‐γ) production by CD8⁺ T cells, suggesting compromised effector function (Figure 4D). Viral load and cell infiltration in peripheral blood and spleen were unaffected, indicating a meninges‐specific defect (Figure S6E–J, Supporting Information). Whole‐mount staining revealed that ST2 deletion reduced meningeal CD8⁺ T cell infiltration into the parenchyma and cluster formation upon infection (Figure 4E,F).

We isolated meningeal mast cells from infected *Tpsb2‐CreERT2; Il1rl1^fl/fl^* mice at multiple time points for SMART‐seq analysis (Figure 4G). Compared to wild‐type, ST2‐deficient mast cells displayed markedly reduced expression of cytokines, chemokines, ISGs, and antigen‐processing and presentation‐related molecules (Figure 4H,I). These transcriptional differences emerged by 4 dpi and progressively intensified, underscoring a critical role for ST2 signaling in mast cell transcriptional activation during infection. ST2 deletion also impaired mast cell degranulation by 6 dpi (Figure 4J), indicating that ST2‐mediated signaling drives transcriptional and functional activation essential for immune cell recruitment and viral clearance.

Finally, impaired viral clearance in the brain parenchyma after secondary LCMV challenge indicated persistent CNS antiviral immunity defects (Figure 4K). In addition, using bacterial (Listeria monocytogenes, Streptococcus pneumoniae) and fungal (Cryptococcus neoformans) meningeal infection models (Figure S6K, Supporting Information), we found that ST2 deletion in mast cells specifically impaired host defense against Listeria monocytogenes but not against other pathogens (Figure 4L). This supports a critical role for IL‐33‐ST2 signaling in mast cells in defense against intracellular pathogens like LCMV and Listeria. We hypothesize that intracellular IL‐33 release triggered by such infections activates ST2 signaling.

### 2.5 IL‐33 Signal from Meningeal Stromal Cells Promotes the Antiviral Immune Response and CD8^+^ T Cell Infiltration

**Figure 5:**
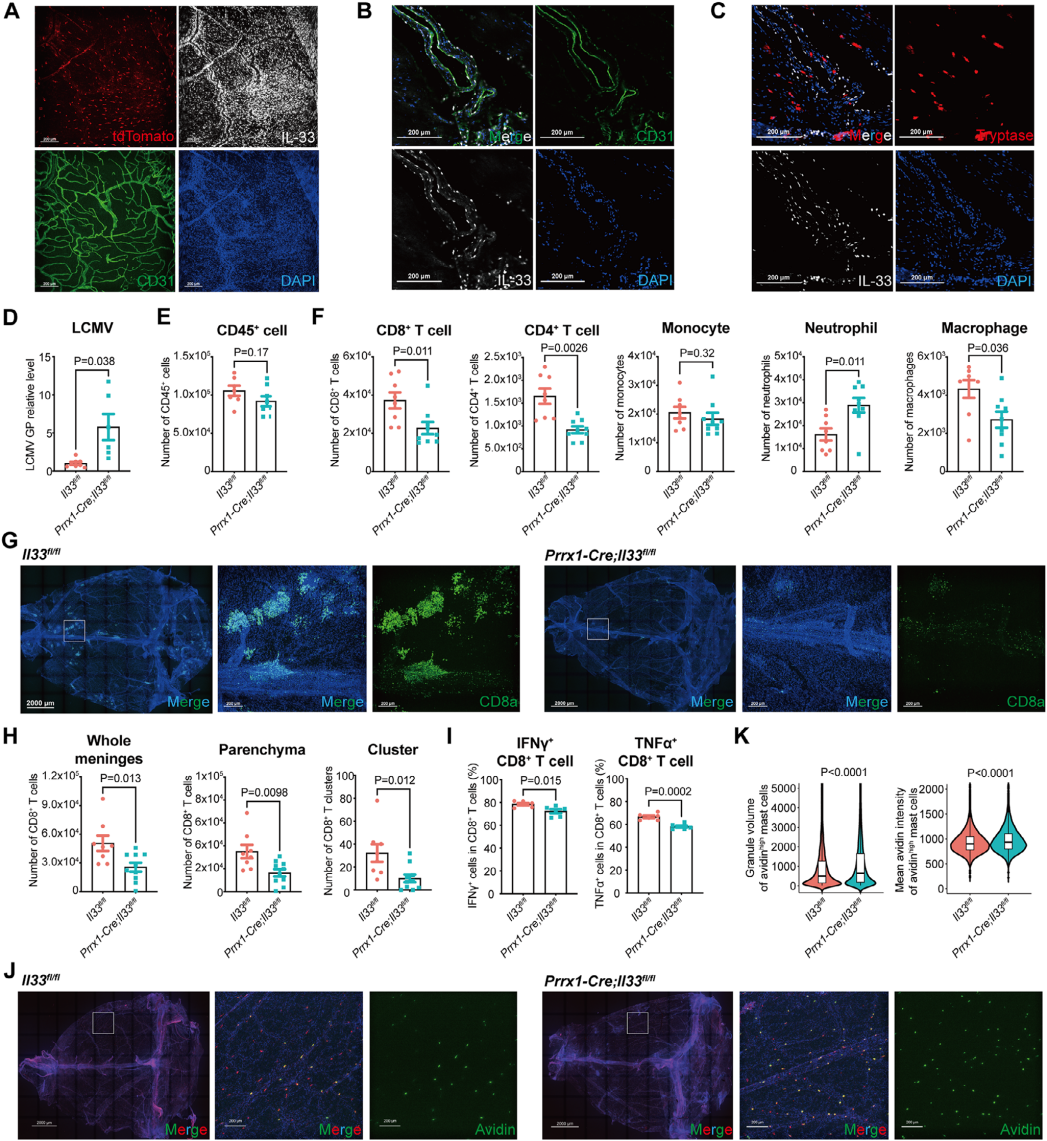
IL‐33 signal from meningeal stromal cells promotes the antiviral immune response and CD8^+^ T cell infiltration. A) Maximal intensity projection of dural meninges stained for mast cells (red), IL‐33 (white), blood vessels (green), and nuclei (blue) from *Tpsb2‐CreERT2; Ai14^fl/+^* mice. n = 6 (mice). B) Spatial distribution of blood vessel (green), IL‐33 (white), and nuclei (blue) stained on continuous slices from adult meninges. C) Spatial distribution of mast cells (red), IL‐33 (white), and nuclei (blue) stained on continuous slices from adult meninges. D‐K) *Prrx1‐Cre; Il33^fl/fl^* mice were used to delete IL‐33 in stromal cell. D) LCMV mRNA level in the dural meninges at 6 dpi. n = 6–8 (mice). E) Meningeal immune cell counts using whole mount CD45 staining at 6 dpi. n = 7–8 (mice). F) Meningeal cell calculated counts using CD45 staining and FACS at 6 dpi. n = 8–9 (mice). G) Maximal intensity projection of dural meninges stained for CD8⁺ T cells (green) and nuclei (blue) at 6 dpi. n = 8–10 (mice). H) The number of CD8⁺ T cells (G). I) The IFN‐γ and TNF‐α production by meningeal CD8⁺ T cells at 6 dpi. n = 5 (one point from 2 mice). J) Maximal intensity projection of dural meninges stained for granule (green), mast cell (red), and nuclei (blue) at 6 dpi. n = 7–9 (mice). K) Granule volume of avidin^high^ mast cells analyzed by volume, mean intensity of avidin^+^ granules (J). n = 19226‐21291 (cells). Scale bar for all images, 200 µm. Data are presented as mean ± SEM and *p* values were calculated by two‐tailed, unpaired Student's *t*‐test (D–F, H–I, K).

The ligand for ST2, IL‐33, is primarily expressed in epithelial and vascular endothelial cells in mucosal tissues.^[^ ^51^, ^52^, ^53^ ^]^ Upon tissue damage, IL‐33 is often released from the nucleus to the extracellular space, mediating tissue remodeling and inflammatory responses. Here, we found that IL‐33 was predominantly expressed in meningeal fibroblasts among stromal cells in mice (Figure S7A, Supporting Information). IL‐33 showed nuclear localization throughout the meninges, with no expression difference noted upon infection (**Figure** **5**A; Figure S7A, Supporting Information). Further, we detected spatial proximity between IL‐33‐expressing stromal cells and mast cells, consistent with potential paracrine signaling to meningeal mast cells (Figure 5A). In human meninges, IL‐33 was also predominantly expressed in stromal cells—though in different subsets, including fibroblasts and vascular endothelial cells—and localized near mast cells, implying conserved IL‐33‐mediated crosstalk (Figure 5B,C; Figure S7B, Supporting Information).

To specifically delete *Il33* in stromal cells, we generated *Prrx1‐Cre* (*Prrx1‐Cre; Il33^fl/fl^*) mice (*Prrx1‐Cre* labeled meningeal stromal cells, Figure S7C–E, Supporting Information). Following LCMV infections, stromal cell‐specific IL‐33 deletion increased meningeal viral loads and reduced CD8⁺ T cells infiltration, as analyzed by RT‐qPCR, immunofluorescence staining, and FACS (Figure 5D–F; Figure S7F, Supporting Information). Decreased CD4⁺ T cell infiltration was also observed. Whole‐mount staining confirmed reduced CD8⁺ T cell recruitment and cluster formation, alongside a significant reduction of IFN‐γ and TNF‐α production by CD8⁺ T cells (Figure 5G–I). Immune cell composition in the spleen or blood remained unaffected, highlighting the meninges‐specific impact (Figure S7G–K, Supporting Information). Moreover, IL‐33 deletion attenuated mast cell degranulation, underscoring the crucial role in activating mast cell effector functions during viral infection (Figure 5J,K; Figure S7L, Supporting Information).

Conversely, in vivo administration of recombinant IL‐33 during infection significantly enhanced meningeal antiviral immune responses, further emphasizing the critical role of IL‐33‐ST2 signaling in mast cell‐mediated immunity (Figure S7M,N, Supporting Information).

Together, these data show that IL‐33‐ST2 signal from meningeal stromal cells to mast cells played an important role for promoting CD8⁺ T recruitment and viral clearance.

### 2.6 Meningeal Stromal Cell‐Derived IL‐33 and ATP Coordinate Antiviral Defense

**Figure 6:**
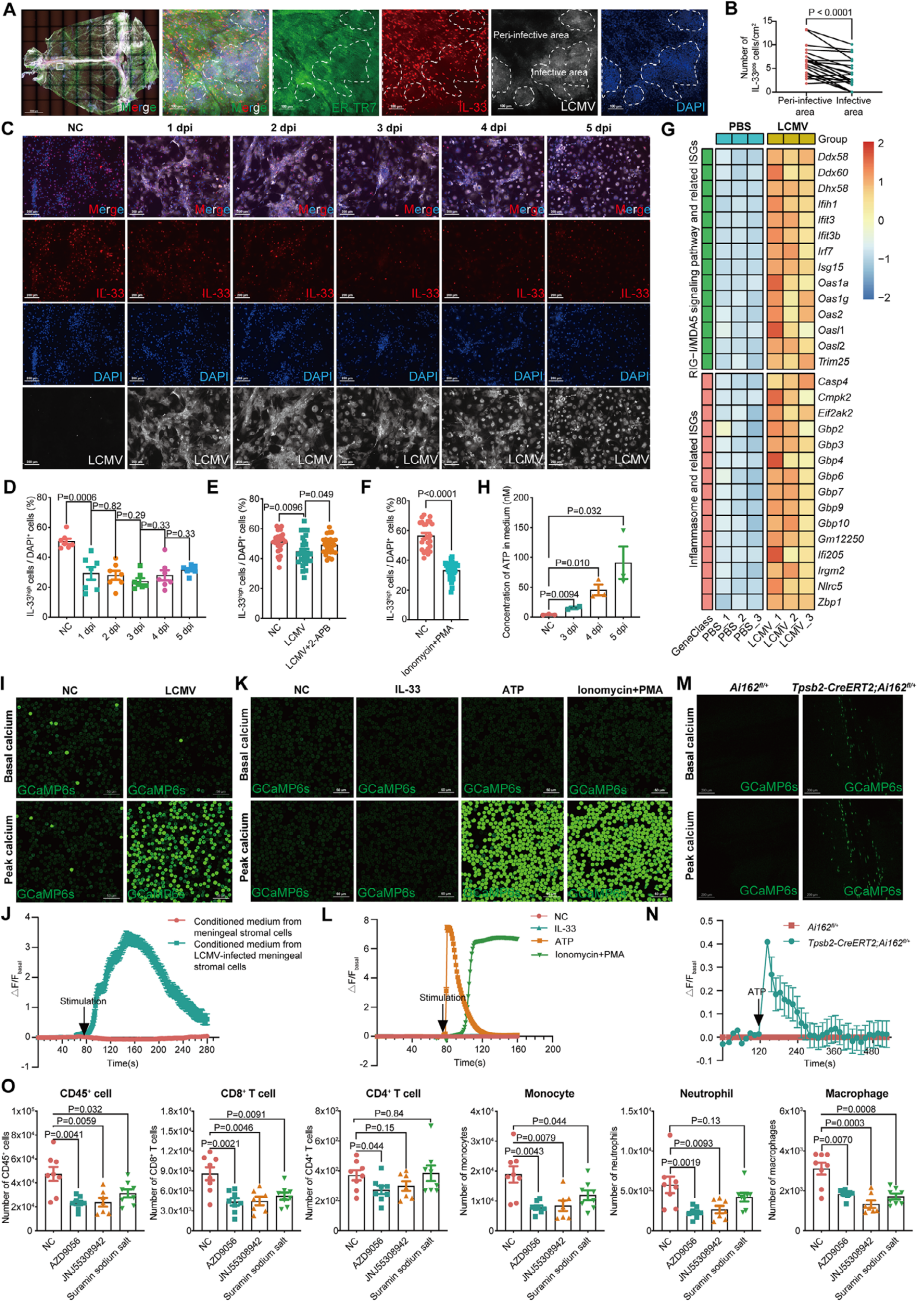
Meningeal stromal cell‐derived IL‐33 and ATP coordinate antiviral defense. A) Maximal intensity projection of dural meninges stained for stromal cells (green), IL‐33 (red), LCMV (white) and nuclei (blue) at 6 dpi. Scale bar, 2000 µm (left, whole mount); 100 µm (right). n = 6 (mice). B) The density of meningeal IL‐33^+^ stromal cells (A). n = 20 (clusters, from 6 mice). C–H) Primary meningeal stromal cells were cultured. C) Staining of IL‐33 (red), nuclei (blue), and LCMV (white) post LCMV infection. MOI = 2:1. Scale bar, 200 µm. n = 6–7 (views). D) The percentage of IL‐33^high^ cells (C). E) The percentage of IL‐33^high^ cells treated with medium alone (NC), LCMV, LCMV + 2‐APB at 1 dpi. MOI = 1:1. n = 25‐28 (views). F) The percentage of IL‐33^high^ cells treated with medium alone (NC), or ionomycin + PMA for 1 day. n = 24‐36 (views). G) Heatmap of differentially expressed genes at 1 dpi. MOI = 2:1. n = 3 (wells, each well from 2 mice). H) The extracellular ATP level after LCMV infection. MOI = 2:1. n = 2‐3 (wells, each well from 2 mice). I) Live cell imaging of calcium flux in PCMCs from *Tpsb2‐CreERT2; Ai162^fl/+^* mice stimulated with conditioned medium from virus‐infected stromal cells. Scale bar, 50 µm. n = 75–275 (cells). J) Quantification of (I). K) Live cell imaging of calcium flux in BMMCs from *Tpsb2‐CreERT2; Ai162^fl/+^* mice stimulated with IL‐33, ATP, or ionomycin + PMA. Scale bar, 50 µm. n = 156‐356 (cells). L) Quantification of (K). M) Live cell imaging of calcium flux in the meninges from *Tpsb2‐CreERT2; Ai162^fl/+^* mice with the stimulation of ATP *ex vivo*. Scale bar, 200 µm. n = 33 (cells). N) Quantification of (M). O) Meningeal immune cell subset counts treated with ATP receptor antagonist (AZD9056, JNJ55308942, suramin sodium salt) at 6 dpi. n = 7–8 (mice). Data are presented as mean ± SEM and *p* values were calculated by two‐tailed, paired Student's *t*‐test (B), two‐tailed, unpaired Student's *t*‐test (D–F, H, O), Wald test from DESeq2 (G).

LCMV tropism analysis identified ER‐TR7‐expressing stromal cells in the meninges as primary targets,^[^ ^54^ ^]^ which exhibited high basal IL‐33 expression (**Figure** **6**A). Viral infection triggered IL‐33 release, indicated by significantly reduced IL‐33 signals specifically within infected meningeal regions (Figure 6A,B). In vitro, primary mouse meningeal stromal cells exhibited rapid intracellular IL‐33 depletion during early infection (Figure 6C,D). Consistent with previous findings suggesting a cellular calcium influx‐induced IL‐33 release during pathogen invasion,^[^ ^55^, ^56^ ^]^ inhibition of calcium influx with 2‐aminoethyl diphenylborinate (2‐APB) attenuated IL‐33 release, whereas activation via ionomycin and phorbol 12‐myristate 13‐acetate (PMA) treatment enhanced it (Figure 6E,F).

Transcriptomic profiling of infected stromal cells revealed upregulation of RIG‐I/MDA5 signaling components, and inflammasome‐related genes (Figure 6G). Despite this innate immune response, viral titers progressively increased until CD8⁺ T cell infiltration at day 6, underscoring the indispensable role of the adaptive immunity in viral clearance. Concurrent induction of pyroptosis‐linked genes (e.g., *Casp4*, *Gbp2*),^[^ ^57^, ^58^, ^59^ ^]^ suggested cellular damage involvement.

Analysis revealed ATP release followed IL‐33 during virus‐induced damage (Figure 6H). ATP acts as a mast cell degranulating factor.^[^ ^36^ ^]^ Conditioned medium from infected stromal cells triggered calcium flux in mast cells (using *Tpsb2‐CreERT2*; *Ai162* reporter mice), an effect recapitulated by ATP but not IL‐33 (Figure 6I–N; Videos S1–S8, Supporting Information). Blocking ATP receptors (P2RX7‐selective antagonists AZD9056 and JNJ55308942, or broad‐spectrum P2X/P2Y antagonist suramin) impaired meningeal antiviral immunity without affecting systemic responses (Figure 6O; Figure S8A–C, Supporting Information). To further investigate the role of P2RX7 on mast cells, we generated mast cell–specific *P2rx7* knockout mice (*Tpsb2‐CreERT2; P2rx7^fl/fl^*). Consistently, following LCMV infection, loss of *P2rx7* led to a significant reduction in meningeal immune cell infiltration (Figure S8D–G, Supporting Information).

In sum, LCMV infection induced the release of IL‐33 and ATP from stromal cells. IL‐33 served as an early alarmin, while ATP augmented mast cell activation, collectively bridging innate and adaptive antiviral responses in the meninges.

### 2.7 IL‐33 Licenses Mast Cells for Potent Activation by ATP

**Figure 7:**
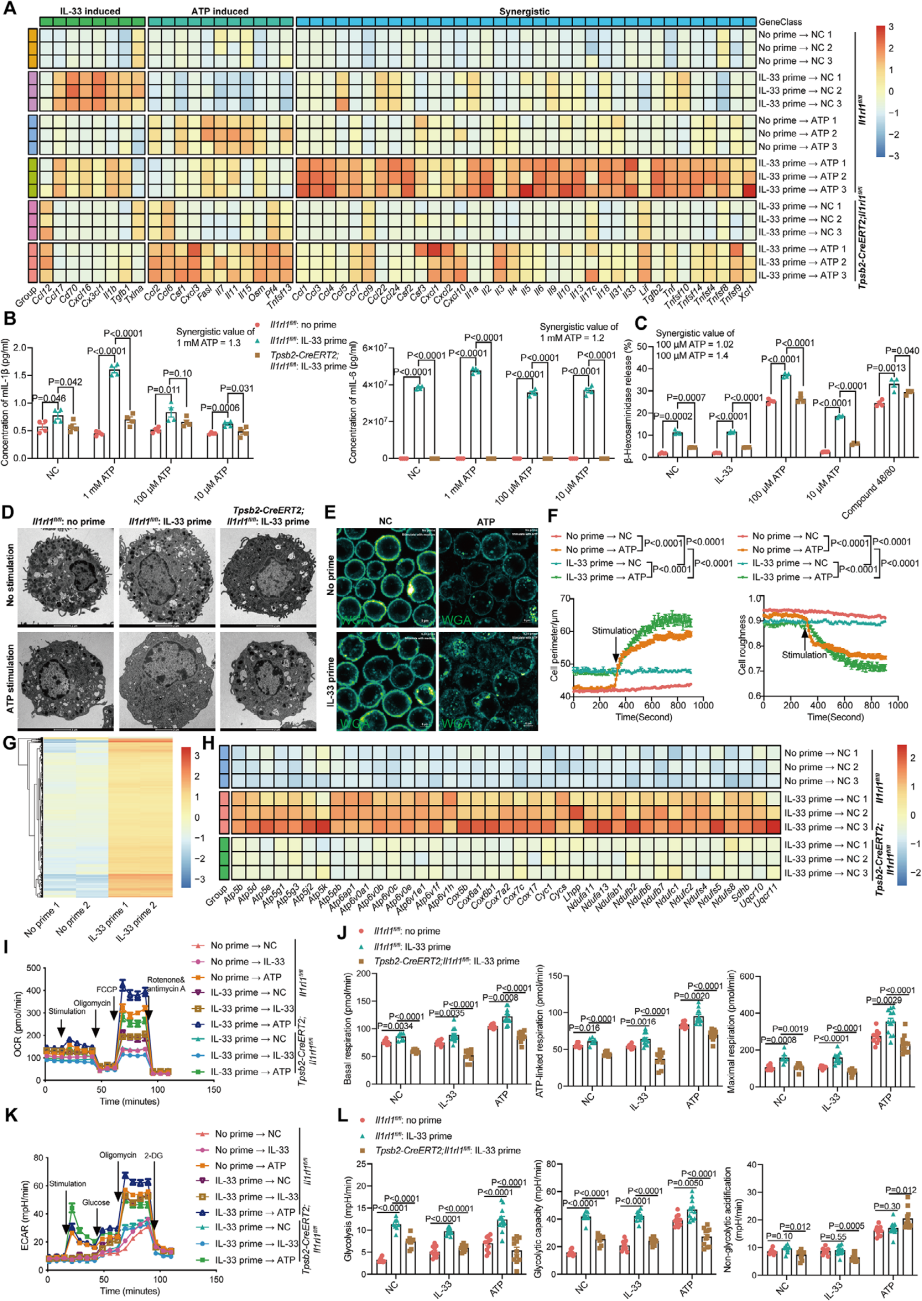
IL‐33 licenses mast cells for potent activation by ATP. A–L) PCMCs from control (*Il1rl1^fl/fl^*) and ST2‐deleted (*Tpsb2‐CreERT2; Il1rl1^fl/fl^*) mice were treated with IL‐33 for 24 h, then stimulated with ATP followed by RNA‐seq (A, H), ELISA (B), degranulation (C–F), ATAC‐seq (G), and seahorse (I–L). A) Heatmap of differentially expressed cytokines and chemokines. n = 3 (wells). B) The protein levels of IL‐1β and IL‐6. n = 4 (wells). C) Degranulation measurement of β‐hexosaminidase release assay. n = 4 (wells). D) Degranulation imaging with transmission electron microscopy. Scale bar, 2 µm. E) Degranulation imaging for real‐time by WGA (green fire). Scale bar, 5 µm. n = 3 (wells). F) Quantification of (E). G) Heatmap of peak RPM by ATAC seq. n = 2 (wells). H) Heatmap of differentially expressed oxidative phosphorylation related genes. n = 3 (wells). I) OCR measurement. n = 6‐12 (wells). J) Quantification of (I). K) ECAR measurement. n = 6–12 (wells). L) Quantification of (K). Data are presented as mean ± SEM and *p* values were calculated by Wald test from DESeq2 (A, H), two‐tailed, unpaired Student's *t*‐test (B, C, J, L), one‐way ANOVA (F).

To model functional effects of IL‐33 and ATP, PCMCs were treated with IL‐33 for 24 h followed by ATP stimulation for 4 h, then subjected to transcriptome sequencing. Dual IL‐33 and ATP stimulation significantly upregulated cytokines and chemokines, with 65% exhibiting synergistic effects (**Figure** **7**A). Key genes such as *Il1β*, *Il6*, *Tnfα*, *Ccl1*, *Ccl4*, and *Ccl7* exhibited dose‐dependent differential expression, critical for CD8⁺ T cell recruitment and activation (Figure S9A–C, Supporting Information). This synergy was transcriptome‐wide, indicating IL‐33 priming exerts greater influence than ATP alone (Figure S9D, Supporting Information). Protein level analysis confirmed IL‐33 priming was pivotal for cytokine and chemokine induction upon subsequent ATP stimulation (Figure 7B).

Notably, IL‐33 alone did not trigger degranulation but significantly enhanced ATP‐induced degranulation (Figure 7C). ATP alone produced minimal extracellular particles, whereas IL‐33 priming markedly increased cell size and particle release, as analyzed by transmission electron microscopy (Figure 7D). IL‐33 priming also substantially increased cell size and reduced surface roughness, visualized via WGA‐based real‐time degranulation imaging method, consistent with enhanced granular content and vesicle‐membrane trafficking upon dual stimulation (Figure 7E,F; Videos S9–S12, Supporting Information).

We first tested whether IL‐33 alters genome accessibility for transcriptional synergy. ATAC‐seq revealed significant chromatin accessibility changes following IL‐33 priming, but not specifically at synergistically upregulated gene loci (Figure 7G; Figure S9E, Supporting Information).

KEGG enrichment analysis of upregulated genes in IL‐33‐primed mast cells identified oxidative phosphorylation‐related pathways among top categories (Figure 7H; Figure S9F, Supporting Information), prompting investigation of metabolic impacts. IL‐33 priming robustly increased glycolysis and significantly enhanced mitochondrial respiration and ATP production, indicating substantial metabolic reprograming to support transcription and degranulation processes (Figure 7I–L; Figure S9G, Supporting Information).

These synergistic effects extended beyond PCMCs to bone marrow‐derived mast cells (BMMCs), suggesting a generalizable mechanism involving coordinated transcriptional, degranulation, and metabolic regulation (Figure S10A–J, Supporting Information).

Wild‐type mice pretreated with recombinant IL‐33 followed by ATP supplementation showed amplified antiviral immunity (Figure S11A, Supporting Information), whereas *Il33* ablation impaired meningeal responses (Figure S11B, Supporting Information), underscoring the role of IL‐33 priming in meningeal antiviral defense. While these findings provide a potential basis for therapeutic development, further studies are required to define appropriate modalities and dosing regimens to achieve localized immunomodulation without systemic consequences.

Moreover, IL‐33 priming synergistically enhanced cytokine and chemokine responses to GPCR‐Gq activation (clozapine/hM3Dq agonist) in PCMCs, significantly amplifying degranulation, indicating broader relevance beyond ATP (Figure S11C–G, Supporting Information).

Finally, single‐cell RNA sequencing revealed that infection‐activated mast cells enhanced communication with CD8⁺ T cells and vascular endothelial cells, suggesting both direct and indirect roles in promoting T‐cell infiltration into the meninges (Figure S3F, Supporting Information). Consistent with this, pharmacological blockade of CCR5 with maraviroc reduced meningeal immune cell recruitment (Figure S12A, Supporting Information). In parallel, we applied supernatants from IL‐33‐primed, ATP‐stimulated mast cells to mouse aortic endothelial cells (MAECs) and bone marrow–derived dendritic cells (BMDCs). This treatment altered gene expression in both cell types: MAECs showed transcriptioal induction of *Vcam1*, *Icam1*, *Sele*, *Il6*, *Ccl2*, and *Cxcl1*, while BMDCs upregulated *Cd80*, *Cd86*, and *Il6* (Figure S12B,C, Supporting Information). Furthermore, pharmacogenetic activation of mast cells enhanced vascular permeability (Figure S12D, Supporting Information). Together, these results indicate that mast cells orchestrated meningeal immunity by producing chemotactic and inflammatory mediators, thereby facilitating CD8⁺ T cell recruitment through both direct and indirect mechanisms.

In conclusion, IL‐33 primes mast cells for potentiated responses to ATP and other ligands (e.g., GPCR agonists), generating synergistic activation across transcriptional, degranulation, and metabolic programs, thereby promoting CD8⁺ T cell infiltration during antiviral defense.

## 3 Discussion

The coordinated response of immune cells within the meninges is pivotal for protective immunity against pathogen invasion. Here, we elucidated the crucial role of mast cells in combating viral infections in the meninges by promoting the recruitment and activation of CD8⁺ T cells. Furthermore, we delineated how IL‐33 derived from stromal cells primed mast cells for effective immune activation. These findings reveal the significant function of mast cells and associated pathways in meningeal immune responses. Investigating the full spectrum of immune subtypes will provide crucial insights into barrier immunity in brain health.

In this study, we established a new mast cell mouse line, *Tpsb2‐CreERT2*, in combination with genetic manipulation strategies, and uncovered an unexpected role for mast cells and the IL‐33/ST2 axis in antiviral immunity within the meninges, challenging the conventional understanding of these components as exclusive mediators of type 2 immunity in parasitic and allergic contexts.^[^ ^51^ ^]^ While the *Tpsb2‐CreERT2* model offers multiple advantages, further characterization is required, such as assessing labeling efficiency across tissues and evaluating potential subtle effects on tryptase β−2 function. Functionally, our findings align with emerging evidence that immune cells and cytokine networks are not strictly confined to type 1 or type 2 immunity but exhibit functional plasticity dictated by their microenvironment.^[^ ^60^, ^61^ ^]^ For instance, mast cells demonstrate remarkable versatility: during bacterial infections, they recruit neutrophils and dendritic cells to clear pathogens or bridge innate and adaptive immunity,^[^ ^34^, ^40^, ^42^ ^]^ while in viral contexts, their roles range from enhancing viral clearance to suppressing immune responses.^[^ ^39^, ^62^, ^63^ ^]^ Such pleiotropy underscores the necessity of defining immune cell functions within precise spatial and pathological settings. Our work highlights the meninges as a critical site where mast cells pivot from classical type 2 roles to engage in antiviral defense, expanding the paradigm of immune cell adaptability.

Our findings further revealed that IL‐33 licensing markedly enhanced the response of meningeal mast cells to subsequent stimulation, enabling rapid and effective antiviral responses that eliminated infection, thereby protecting the brain parenchyma. Consistent with this observation, Mamuladze et al. recently showed that mast cell degranulation in the meninges can rapidly regulate CSF drainage via the ACE point, thereby restricting pathogen entry into the parenchyma.^[^ ^29^ ^]^ Although these mechanisms differ—IL‐33 acting through immunological licensing and degranulation regulating CSF dynamics—they converge on a shared outcome: limiting pathogen invasion into the brain. Moreover, since IL‐33 priming also potentiates mast cell degranulation, it is reasonable that enhanced degranulation not only amplifies immune responses but also contributes to CSF regulation, spatially confining pathogens and inflammation to the meninges. Although we did not directly investigate IL‐33/ST2 regulation of CSF dynamics, this represents an intriguing direction for future studies.

The implications of meningeal immune activity extend beyond pathogen clearance to brain homeostasis. Viral infections and meningeal inflammation are linked to behavioral changes, though their interplay remains poorly defined. Intriguingly, mast cells have been implicated in neurological conditions such as stroke and migraine, including alcohol‐withdrawal‐associated headaches.^[^ ^24^, ^64^, ^65^ ^]^ While our focus was on viral defense, mast cell activation in the meninges may reshape the local immune milieu, potentially influencing anxiety, cognition, and neuropathology—both during infection and in sterile contexts.^[^ ^66^, ^67^ ^]^ This raises critical questions: Does meningeal mast cell activity contribute to the neurological sequelae of infections? How do their mediators intersect with neural circuits? Resolving these connections could reveal novel therapeutic targets for brain disorders.

Moreover, meningeal immunity is not isolated; it interfaces dynamically with peripheral systems. For example, gut‐derived signals via the “gut‐meningeal axis” modulate antifungal immunity, suggesting that mast cells—heterogeneous across tissues—may serve as sentinels integrating systemic cues with CNS homeostasis.^[^ ^68^ ^]^ Peripheral inflammation or dysbiosis could thereby perturb meningeal mast cells, exacerbating or mitigating neuropathology. Future studies dissecting how tissue‐specific microenvironments and inter‐organ communication shape mast cell phenotypes will be vital for designing CNS‐focused therapies.

Within the meninges, mast cells coexist with various immune cell populations, including B cells critical for CNS immune tolerance. Mast cells secrete cytokines such as IL‐4 and IL‐6, which may influence B cell development or autoreactivity, yet their interplay in autoimmune diseases remains unexplored. Similarly, mast cell‐macrophage crosstalk in the meninges could regulate immune responses, particularly given the prevalence of macrophages in this compartment. Understanding these interactions could help uncover mechanisms underlying CNS‐associated diseases.

Mechanistically, key questions persist. Although MHC‐I antigen processing and presentation pathways were activated in meningeal mast cells following infection and exhibited dependence on ST2 signaling, their antigen‐presenting capacity requires further investigation. While we observed a reduction of IL‐33 in infected stromal cells via nuclear staining, we were unable to detect free IL‐33 in vitro or in vivo. This suggests additional analyses are warranted to understand this process; for instance, whether a distinct, undetectable form of IL‐33 by current kits or methods is present. The pathogen selectivity of IL‐33 signaling suggests that different pathogens may differentially trigger IL‐33 release. The precise mechanisms remain to be elucidated, but may depend on stromal cell susceptibility and the signaling pathways they engage by intracellular versus extracellular microbes.^[^ ^69^ ^]^ In mast cells, multi‐omics analyses revealed that IL‐33 priming induced synergistic activation across transcription, translation, and degranulation, with metabolic rewiring providing a potential mechanistic basis. However, whether metabolic changes act as a driver of functional shifts remains unclear.^[^ ^70^, ^71^, ^72^ ^]^ For example, does inhibition of IL‐33–induced metabolic enhancement directly impair cytokine production by limiting energy availability, or indirectly by modulating the abundance of the transcriptional and translational machinery? Elucidating these pathways will be critical to understanding how meningeal immunity balances host defense with tissue protection.

Mast cells, as multifaceted immune cells, can also drive pathology when aberrantly activated. In stroke, meningeal mast cell activation exacerbates tissue damage, while mast cell deficiency reduces infarct size and inflammation.^[^ ^27^, ^28^ ^]^ In migraine, reciprocal activation between mast cells and nociceptors is central to disease onset, and inhibition of mast cell activation has been proposed as a therapeutic strategy.^[^ ^22^ ^]^ Mast cells have also been implicated in Alzheimer's disease–associated cognitive decline.^[^ ^66^, ^73^ ^]^ Excessive mast cell activation in allergic or infectious settings can cause tissue injury and, in severe cases, fatal shock.^[^ ^74^ ^]^ Furthermore, mast cell–driven angiogenesis supports tumor progression.^[^ ^75^ ^]^ Collectively, these findings emphasize that mast cell activity cannot be simply categorized as beneficial or detrimental; instead, their roles must be evaluated within specific tissue microenvironments and disease contexts.

CD8⁺ T cells similarly display dual roles in CNS viral infections. In LCMV‐induced meningitis, whether they confer protection or pathology depends on infection route, viral dose, and the extent of parenchymal infiltration.^[^ ^76^ ^]^ In our tail vein injection model using a non‐lethal viral dose, meningeal immune responses were robust while parenchymal involvement remained limited, indicating a protective role for mast cell–mediated CD8⁺ T cell activation. At the same time, prior studies have clearly demonstrated pathogenic CD8⁺ T cell–driven immunopathology in the CNS.^[^ ^77^, ^78^ ^]^ Together, these findings highlight the importance of context in determining CD8⁺ T cell function.

Activation of meningeal mast cells promoted antiviral immunity, with mechanistic analyses identifying IL‐33/ST2 signaling as a key pathway. Pretreatment with IL‐33 primed mast cells into a heightened state of responsiveness, enabling more efficient responses to subsequent signals. These results suggest that locally activating mast cells or delivering IL‐33 to the meninges could enhance CNS resistance to infection. Conversely, mast cell stabilizers and chronic blockade of IL‐33, widely used for treating type 2 immune disorders such as asthma and dermatitis, may inadvertently increase susceptibility to pathogens.^[^ ^79^, ^80^ ^]^ Supporting this concept, Mamuladze et al. demonstrated that histamine treatment provides protective effects in bacterial meningitis, underscoring the therapeutic potential of modulating mast cell pathways in CNS infections.^[^ ^29^ ^]^

In summary, our study redefines mast cells as versatile players in meningeal antiviral immunity, mediated through stromal cell crosstalk (Figure S12E, Supporting Information). These insights broaden the conceptual framework of CNS barrier immunity and underscore the therapeutic potential of targeting mast cell pathways in infections and neuroinflammatory disorders. Future work dissecting the molecular and systemic networks governing meningeal immunity will be essential for harnessing these cells in brain health and disease.

## 4 Experimental Section

### Animal Information

All surgical and experimental procedures in mice were performed in compliance with the protocol approved by the Institutional Animal Care and Use Committee (IACUC) of Tsinghua University.

The animals were housed under a 12‐h light/12‐h dark cycle (lights on from 07:00 to 19:00), with temperature maintained between 20–23 °C and relative humidity between 40–65%. They were provided with ad libitum access to chow diet and water. Wild‐type and mutant mice were bred in‐house to generate littermates for experimentation. Each cage housed no more than 6 mice, ranging from weanlings to adults. Mice were grouped according to genotype, with all experimental groups for animal studies being age‐matched, numbered, and randomly assigned to different treatment groups. Measurements for all animal experiments were conducted in a blinded manner. Sample size calculations were not performed before in vivo experiments.

Male and female mice aged 2 to 37 weeks, maintained under specific pathogen‐free conditions, were utilized in the experiments. With the exception of optogenetic experiments, all other experiments shown here were conducted using female mice and male mice tended to show less obvious phenotypic differences. Wild‐type C57BL/6J mice were procured from Charles River International. *Ai14* (JAX 007908, RRID:IMSR_JAX:007908), *Ai32* (JAX 012569, RRID: IMSR_JAX:012569), *Prrx1‐Cre* (JAX 005584, RRID:IMSR_JAX:005584), *Ai162* (JAX 031562, RRID:IMSR_JAX:031562), *hM3Dq* (JAX 026220, RRID:IMSR_JAX:026220) were from the Jackson Laboratory. *Cpa3‐CreERT2* (NM‐KI‐200005) mice were obtained from Shanghai Model Organisms Center. *Cpa3‐Cre* (I001146) mice were obtained from Cyagen Biosciences. *P2rx7^fl/+^* (T009615) mice were obtained from GemPharmatech. The *DTA* mice were kindly provided by Professors Zhongjun Dong and Yeguang Chen.

### Generation of Tpsb2‐CreERT2 Mice

The *Tpsb2‐CreERT2* mouse line was established by inserting a cassette before the stop codon of the *Tpsb2* gene using a targeting vector. This targeting vector was designed with a P2A site followed by the iCreERT2 sequence immediately preceding the stop codon of the *Tpsb2* coding region. The linearized targeting vector, along with Cas9 mRNA and sgRNA (5′‐ GTCTAAGTAGTATGTCACCC‐3′), was microinjected into C57BL/6 mouse zygotes. Offspring resulting from this procedure underwent screening via PCR genotyping, DNA sequencing, and Southern blot analysis to identify the targeted mice. Subsequently, these mice were bred with *Ai14* and *Ai32* reporter mice to facilitate flow cytometric and immunofluorescent analyses of cell‐specific recombination.

To induce genetic recombination specifically in mast cells, tamoxifen (Macklin, T832955) dissolved in corn oil (100 mg kg^−1^) was administered via intraperitoneal injection to mice of the indicated genotype daily for 3 days. Subsequently, the mice were analyzed 7 days post‐treatment, and the recombination efficiency in mast cells reached nearly 100%.

For mast cell depletion using *Tpsb2‐CreERT2; DTA^fl/fl^* mice, tamoxifen was administered intraperitoneally for 4 consecutive days, followed by a 3‐day rest, and then another 3‐day course of daily injections. Experiments were performed 4 days after the second injection cycle, and mast cell depletion efficiency reached ≈90%.

### Generation of Il1rl1^fl/fl^ Mice

The *Il1rl1^fl/fl^* mouse line was established by inserting two loxP sites. One loxP site was inserted to the intron 2 between exon 2 and exon3. The other loxP site was inserted to the intron 4 between exon 4 and exon 5. The linearized targeting vector, along with Cas9 mRNA and sgRNA (5′‐sgRNA1: 5′‐ GTGGTCACCAAACCATAGGG ‐3′; 3′‐sgRNA2: 5′‐ AGACCCCATGTCTTCGATAG ‐3′), was microinjected into C57BL/6 mouse zygotes. Offspring resulting from this procedure underwent screening via PCR genotyping, DNA sequencing, and Southern blot analysis to identify the targeted mice. Subsequently, these mice were bred with *Tpsb2‐CreERT2* mice to facilitate RNA analysis of recombination.

### Generation of Il33^fl/fl^ Mice

The *Il33^fl/fl^* mouse line was established by inserting two loxP sites. One loxP site was inserted to the intron between exon 2 and exon3. The other loxP site was inserted to the intron between exon 7 and exon 8. The linearized targeting vector, along with Cas9 mRNA and sgRNA (5′‐sgRNA1: 5′‐ AACCACTCAAGGCATTCGTG ‐3′; 3′‐sgRNA2: 5′‐ GCTCTCGCTCCAAGACAGTC ‐3′), was microinjected into C57BL/6 mouse zygotes. Offspring resulting from this procedure underwent screening via PCR genotyping, DNA sequencing, and Southern blot analysis to identify the targeted mice. Subsequently, these mice were bred with *Prrx1‐Cre* mice to facilitate RNA analysis of recombination.

### Pathogen‐Induced Meningitis

LCMV Armstrong was kindly provided by Dr. Lilin Ye and was prepared according to established protocols as described in previous reports.^[^ ^81^ ^]^ The model for LCMV induced meningitis was established by intravenously injecting each mouse with either 1, 2 or 4×10^5 PFU. The model for *Listeria monocytogenes* induced meningitis was established by intracisternal injecting each mouse with 1×10^5 CFU.^[^ ^82^ ^]^ The model for *Streptococcus pneumoniae* induced meningitis was established by intracisternal injecting each mouse with 8×10^4 CFU.^[^ ^83^ ^]^ The model for *Cryptococcus neoformans* induced meningitis was established by intracisternal injecting each mouse with 5×10^4 CFU.^[^ ^84^ ^]^ Pathogen experimental procedures were conducted within the Biosafety Level II Laboratory at Tsinghua University.

### Human Samples

The human meninges were obtained from the Affiliated Hospital of Zunyi Medical University. The study was conducted in compliance with the ethical guidelines of the US Common Rule, and was approved by the Institutional Ethics Committee of the Affiliated Hospital of Zunyi Medical University (KLLY‐2023‐079) with written informed consent obtained from the patients. Meninges were collected from 2 patients (aged 9 and 52 years) undergoing cerebral infarction surgery, followed by immunostaining procedures.

### Secondary Intracranial LCMV Challenge

The meningitis model was initially established by intravenous injection of 2 × 10^5 PFU per mouse. 40 days later, mice were re‐challenged via intracisternal injection with 10000 PFU per mouse. Cortical tissues were collected at 2 or 4 days after secondary injection for viral titer quantification.

### Pharmacological Treatment

For IL‐33 stimulation alone, 150 µg kg^−1^ of IL‐33 (PeproTech, 210–33) was administered via intracisternal injection on days 2 and 4 post‐infection. For the combined IL‐33 and ATP stimulation experiment, 150 µg kg^−1^ of IL‐33 was similarly administered intracisternally on days 2 and 4 post‐infection, followed by intraperitoneal injection of ATP (Sigma, A2383) at 50 mg kg^−1^ on days 5 and 6 post‐infection. For ATP stimulation in IL‐33 knockout mice, ATP was administered intraperitoneally at a dose of 50 mg kg^−1^ on days 5 and 6 post‐infection. For ATP receptor blockade experiments, mice received intraperitoneal injections of 12.5 mg kg^−1^ AZD9056 (MCE, HY‐19427A), 12.5 mg kg^−1^ JNJ‐55308942 (MCE, HY‐123857), or 20 mg kg^−1^ suramin (MCE, HY‐B0879A) on days 1, 3, and 5 post‐infection. For CCR5 blockage experiments, mice received intraperitoneal injections of 25 mg kg^−1^ maraviroc (TargetMol, T6016) daily post‐infection.

### Pharmacogenetic Manipulation

For validation experiments, Clozapine N‐oxide (CNO; 1 mg kg^−1^, WuXi AppTec, ET20041‐1‐P1) was intraperitoneally administered to activate hM3Dq, and tissues were collected 1 h later for analysis. For LCMV infection experiments, CNO was intraperitoneally administered on days 3 and 5 post‐infection to activate hM3Dq, followed by tissue collection on day 6 for analysis. For vascular permeability assays, mice were intraperitoneally injected with clozapine (0.1 mg kg^−1^, Macklin, C831494); 30 min later, 70 kDa Dextran‐RBITC (100 mg kg^−1^, Sigma, R9379) was administered via tail vein injection, and tissues were collected 1 h thereafter for analysis.

### Optogenetic Manipulation

Following anesthesia induction with avertin and subcutaneous injection of meloxicam for analgesia, the mice underwent head hair shaving. Triple antibiotic eye ointment was applied to safeguard the eyes, followed by skin disinfection using iodophor and alcohol. Using surgical scissors, the skin above the target area was incised to expose the skull, which was then thinned for optogenetic micromodule implantation controlled by a microcontroller. Light stimulation in alternating periods of 2 s of illumination with 25 ms pulses at 20 Hz for 1.5 h was performed, with ongoing monitoring of mouse condition.^[^ ^85^ ^]^ Upon completion of stimulation, skin incisions were sutured using 5‐0 nylon, and mice were returned for recovery, with the option of assistance through heating to 37 degrees if required.

### Whole‐Tissue Toluidine Blue Staining of Mouse Meninges and Imaging

Mice were euthanized using CO_2_, followed by the harvesting of the dural meninges, which was then fixed in 1% PFA at room temperature for 1 h. Following fixation, the samples were washed three times with phosphate buffer solution, each wash lasting for 10 min. Toluidine blue staining was conducted according to previously reported methods.^[^ ^7^ ^]^ In brief, the meninges were stained with a 0.25% toluidine blue O (Yuanye, S19057) acid solution (pH 2.3) for 30 min, followed by three washes with double‐distilled water, each lasting for 2 min. Subsequently, the entire meninges were sealed with nail polish. Whole tissue imaging was performed using a Nikon HD25 confocal microscope in brightfield mode.

### Whole‐Tissue Immunofluorescence Staining And Imaging Of Mouse Meninges

Mice were euthanized with CO_2_, followed by harvesting and fixation of the dural meninges using 1% PFA at room temperature for 1 h. The fixed tissue was then washed three times with PBS for 10 min each. Subsequently, the tissues were permeabilized with PBS+0.5% Tween20 for 30 min, followed by PBS+0.2% Tween20+0.3 m glycine for 15 min, both at room temperature. Blocking was performed with PBS+0.2% Tween20 containing 5% FBS for 1 h at room temperature. For immunostaining, the tissue was incubated overnight with the primary antibody in PBS+0.2% Tween20+5% FBS. After washing three times with PBS+0.2% Tween20 at room temperature for 10 min each, the tissue was stained with secondary antibodies in PBS+0.2% Tween20+5% FBS for 2 h at room temperature. Following staining, the tissue was washed three times and mounted.

Imaging of the whole tissue was carried out using a Nikon A1R HD25 confocal microscope, and subsequent data processing, including counting or statistics, was performed using NIS‐Element or Imaris.

The following reagents were used in staining:

Avidin‐FITC (BioLegend, Cat# 405102)

Avidin‐Sulforhodamine 101 (Sigma, Cat# A2348)

WGA‐488 (Thermo Fisher Scientific, Cat# w11261)

WGA‐647 (Thermo Fisher Scientific, Cat# w32466)

The following antibodies were used:

Goat anti‐human/mouse CD117/Kit (R&D, Cat# AF1356, RRID: AB_354750)

Rabbit anti‐Tryptase (Cell Signaling Technology, Cat#: #51550, RRID:AB_3696668)

Rabbit anti‐human mast cell Tryptase (Abcam, Cat# ab151757, RRID:AB_2909419)

Rabbit anti‐human CD31 (Abcam, Cat# ab28364, RRID:AB_726362)

Rabbit anti‐RFP/tdTomato (Rockland, Cat# 600‐401‐379, RRID:AB_2209751)

Rat anti‐CD206‐BV421 (BioLegend, Cat# 141717, RRID:AB_2562232)

Rat anti‐CD8a‐APC (Thermo Fisher Scientific, Cat# 17‐0081‐82, RRID:AB_469335)

Rat anti‐CD8a‐FITC (BioLegend, Cat# 100706, RRID:AB_312745)

Rat anti‐Ly6G‐APC (Thermo Fisher Scientific, Cat# 17‐9668‐82, RRID:AB_2573307)

Rat anti‐Ly6C‐APC/Cy7 (BioLegend, Cat# 128026, RRID:AB_10640120)

Rat anti‐mCD31 (BD Biosciences, Cat# 553370, RRID:AB_394816)

Chicken anti‐GFP (Aves Labs, Cat# GFP‐1020, RRID:AB_10000240)

Rabbit anti‐mUCH‐L1 (Proteintech, Cat# 14730‐1‐AP, RRID:AB_2210497)

Rat anti‐LCMV NP (Bio X Cell, Cat# BE0106, RRID:AB_10949017)

Rat anti‐CD45‐PE (Thermo Fisher Scientific, Cat# 12‐0451‐82, RRID:AB_465668)

Rat anti‐ER‐TR7 (Abcam, Cat# ab51824, RRID:AB_881651)

Mouse anti‐LCMV (Santa Cruz Biotechnology, Cat# sc‐57894, RRID:AB_784381)

Goat anti mIL‐33 (R&D Systems, Cat# AF3626, RRID:AB_884269)

Goat anti hIL‐33 (R&D Systems, Cat# AF3625, RRID: AB_1151900)

### Immunofluorescence Staining and Imaging of Human Meninges

The meningeal samples of patients were collected and fixed in 1% PFA at room temperature for 1 h, followed by three washes with PBS, each lasting 10 min. Subsequently, the samples were dehydrated using a 30% sucrose solution, followed by embedded in OCT:30% sucrose = 2:1 solution. The tissue was then frozen in OCT solution for 15 µm section. The staining and imaging procedures followed the protocols established for mouse meningeal samples.

### Single Cell Suspension Preparation

After euthanizing the mice, the meninges and spleens were carefully collected in cold RPMI 1640 medium, while peritoneal lavage fluid was collected in cold FACS buffer (PBS + 2% FBS + 5 mM EDTA), dorsal skin was collected to HBSS, and peripheral blood was drawn into anticoagulant tubes containing EDTA.

The mouse meninges underwent digestion with 1 mg mL^−1^ collagenase D (Roche, 11088866001) or 0.25 mg mL^−1^ liberase TL (Roche, 5401020001) and 50 µg mL^−1^ DNase I (Roche, 10104159001) at 37 °C for 30 min, followed by filtration through a 70 µm strainer to obtain a single‐cell suspension, which was then transferred to FACS buffer. For the skin, tissues underwent digestion with 1 mg mL^−1^ collagenase IV (Sigma, C4‐28‐100MG) and 100 µg mL^−1^ DNase I (Roche, 10104159001) at 37 °C for 30 min, followed by filtration through a 70 µm strainer to obtain a single‐cell suspension, which was then transferred to FACS buffer. For the spleen, a physical extrusion method was employed to obtain a single‐cell suspension, followed by lysis of erythrocytes with Ack solution. The resulting suspension was then transferred to FACS buffer. As for peripheral blood, erythrocytes were lysed using Ack solution before transferring the single‐cell suspension to FACS buffer.

### Flow Cytometry Analysis

The single‐cell suspension was initially blocked with anti‐CD16/32 antibody and incubated on ice for 30 min. Following washing, surface antigen staining was carried out on ice for 30 min in the dark. Subsequently, 7AAD was utilized for dead and live cell staining, and direct analysis was conducted.

The following reagents were used:

7AAD (Thermo Fisher Scientific, Cat# 00699350)

The following flow cytometry antibodies were used:

Rat anti‐CD45‐eF450 (Thermo Fisher Scientific, Cat# 48‐0451‐82, RRID:AB_1518806)

Rat anti‐CD45‐AF700 (Thermo Fisher Scientific, Cat# 56‐0451‐82, RRID:AB_891454)

Rat anti‐CD117‐FITC (BioLegend, Cat# 105806, RRID:AB_313214)

Armenian hamster anti‐FcεRIα‐APC (Thermo Fisher Scientific, Cat# 17‐5898‐82, RRID:AB_10718824)

Rat anti‐CD11b‐BV510 (BioLegend, Cat# 101263, RRID:AB_2561390)

Rat anti‐CD19‐APC/Cy7 (BioLegend, Cat# 115530, RRID:AB_830707)

Armenian hamster anti‐CD3ε‐FITC (BioLegend, Cat# 100306, RRID:AB_312671)

Rat anti‐CD117‐PE/Cy7 (BioLegend, Cat# 105814, RRID:AB_313223)

Rat anti‐CD117‐PE (BioLegend, Cat# 105808, RRID:AB_313216)

CD16/32 (BioLegend, Cat# 101302, RRID:AB_312800)

Rat anti‐CD8a‐APC (Thermo Fisher Scientific, Cat# 17‐0081‐82, RRID:AB_469335)

Rat anti‐CD8a‐FITC (BioLegend, Cat# 100706, RRID:AB_312745)

Rat anti‐CD8a‐eF450 (Thermo Fisher Scientific, Cat# 48‐0081‐82, RRID:AB_1272198)

Rat anti‐CD4‐APC (BioLegend, Cat# 100412, RRID:AB_312696)

Rat anti‐Ly6C‐APC/Cy7 (BioLegend, Cat# 128026, RRID:AB_10640120)

Rat anti‐Ly6G‐PE (BioLegend, Cat# 127608, RRID:AB_1186104)

Rat anti‐B220‐PE/Cy7 (BD Biosciences, Cat# 552772, RRID:AB_394458)

Rat anti‐IFN‐γ‐PE/Cy7 (BioLegend, Cat# 505826, RRID:AB_2295770)

Rat anti‐TNF‐α‐APC (BioLegend, Cat# 506308, RRID:AB_315429)

### CD8^+^ T Cell Stimulation and Analysis

Single‐cell suspensions were initially treated with protein transport inhibitors monensin (BioLegend, 420701) and brefeldin A (BioLegend, 420601), followed by stimulation with 1 µg mL^−1^ ionomycin (Aladdin, I133497) and 20 ng mL^−1^ PMA (Selleck, S7791). After 4 h, the cells were harvested and underwent sequential blocking, surface antigen staining, fixation, membrane permeabilization, intracellular antigen staining, and were ultimately analyzed.

### Single‐Cell Sequencing and Analysis

After harvesting, the meninges of 10 mice from different groups were washed in ice‐cold RPMI1640 and dissociated using Collagenase IV (Sigma, C5138‐500MG), Collagenase II (Sigma, V900892‐100MG), Collagenase I (Sigma, V900891‐100MG), and DNase I (Sigma, 9003‐98‐9). Cell count and viability were analyzed using a fluorescence Cell Analyzer (Countstar, Rigel S2) with AO/PI reagent after removing erythrocytes (Solarbio, R1010). The debris and dead cells were removed (Miltenyi, 130‐109‐398/130‐090‐101). Finally, fresh cells were washed twice in RPMI 1640 and resuspended at a concentration of 1×10^6^ cells per mL in 1×PBS containing 0.04% bovine serum albumin.

Single‐cell RNA‐Seq libraries were prepared using the SeekOne Digital Droplet Single Cell 5′ library preparation kit (SeekGene, Cat# K00501). Briefly, an appropriate number of cells were mixed with reverse transcription reagent and added to the sample well in the SeekOne DD Chip S3. Barcoded Hydrogel Beads (BHBs) and partitioning oil were dispensed into corresponding wells separately in Chip S3. After emulsion droplet generation, reverse transcription was performed at 42 °C for 90 min and inactivated at 85 °C for 5 min. Next, cDNA was purified from broken droplets and amplified in a PCR reaction. The amplified cDNA product was then cleaned, fragmented, end‐repaired, A‐tailed, and ligated to sequencing adaptors. Finally, indexed PCR was performed to amplify cDNA representing 5′ expression genes, which contained Cell Barcode and Unique Molecular Index. The indexed sequencing libraries were cleaned up with SPRI beads, quantified by quantitative PCR (KAPA Biosystems, Cat# KK4824), and then sequenced on an Illumina NovaSeq 6000 with PE150 read length.

The raw sequencing data underwent initial processing using Fastp to trim primer sequences and remove low‐quality bases.^[^ ^86^ ^]^ SeekOneTools were employed for sequence data processing and alignment to the mouse GRCm38 reference genome, resulting in the generation of gene expression matrices. Subsequently, we utilized Seurat (version 4.3.0) in R (version 4.0.4) to filter out low‐quality cells.^[^ ^87^ ^]^ Cells (PBS group, 1435; LCMV group, 684) with fewer than 350 or more than 7000 detected genes, less than 500 detected counts, or a percentage of mitochondrial genes greater than 2 were excluded. Potential doublets were identified and removed using DoubletFinder (DoubletRate = CellNumber × 8 × 10^−6^).^[^ ^88^ ^]^ The remaining cells (PBS group, 6623; LCMV group, 20857) were then used for subsequent analyses.

Following quality control and filtering, library‐size normalization was performed for each cell using NormalizeData. The top 2000 variable genes were identified using FindVariableFeatures. Subsequently, all libraries were integrated using FindIntegrationAnchors and IntegrateData with default CCA parameters, followed by regression using ScaleData.^[^ ^89^ ^]^ Principal Component Analysis (PCA) and t‐distributed Stochastic Neighbor Embedding (t‐SNE) were employed for dimensionality reduction. FindClusters was utilized to cluster cells using 30 dimensions at a resolution of 2. Cluster annotation was performed manually based on previously reported marker genes.

Differential expression analysis of clustered genes was conducted using FindMarkers, with significance determined by the p value less than 0.05 and the log_2_(fold change) greater than 0.5 following LCMV infection. Mast cell‐specific genes were considered significant by FindMarkers if the adjusted p value was less than 0.05 and the log_2_(fold change) was greater than 0.5 after LCMV infection. Visualization of the results was performed using DimPlot, DotPlot, DoHeatmap, and VlnPlot. The cell to cell commutation was completed by CellChat (v1.6.1) in R.

Single‐cell RNA sequencing data for mouse meninges, peritoneal cells, and gut were obtained from GSE144175 (GSM4904370, GSM4904371), GSE128003 (GSM3660065 – GSM3660158), and GSE124880, respectively.^[^ ^43^, ^44^, ^45^ ^]^ Gene expression levels were reanalyzed using data filtered by nFeature between 350 to 7000 and a percentage of mitochondrial genes less than 5. Clustering was performed with a resolution of 1 and a dimensionality reduction of 1:15. The *Cpa3* ^+^ cluster represented mast cells from different tissues, visualized using DotPlot.

Single‐cell RNA sequencing data for human meninges were obtained from the Zenodo platform (https://doi.org/10.5281/zenodo.4932158).^[^ ^90^ ^]^ RDS documents were imported into R and visualized using VlnPlot.

### SMART‐Seq Sequencing and Analysis

The cells (<200 cells) were transferred into a specialized lysis solution and proceed with cell lysis using PCR machine. Reverse transcription and SMART‐Seq II amplification technology were utilized to synthesize first‐strand cDNA. Library construction was employed the transposase method. DNA nanoballs (DNBs) and combined probe anchored polymerization technology (cPAS) were used for PE100 sequencing.

The raw data obtained by sequencing was filtered using SOAPnuke (v1.5.6) to generate clean data.^[^ ^91^ ^]^ The clean data was aligned to the reference genome using HISAT2 (v2.1.0) and to the reference gene set using Bowtie2 (v2.3.4.3).^[^ ^92^, ^93^ ^]^ RSEM (v1.3.1) was used for gene expression quantification, and DESeq2 (v1.4.5) was used for differential gene detection.^[^ ^94^, ^95^ ^]^ For the experiment of sorted meningeal mast cells using *Tpsb‐CreERT2; Ai14* mice and the experiment of cultured meningeal stromal cells, differential expressed genes were detection with the standard of p value < 0.05 and log_2_FoldChange >0.5. For the experiment of sorted peritoneal mast cell using *Tpsb‐CreERT2; Ai14* mice and the experiment of sorted meningeal mast cell using *Tpsb‐CreERT2; Il1rl1^fl/fl^* mice, differential expressed genes were detection with the only standard of adjusted p value <0.05. The heatmap was completed by pheatmap (v1.0.12) in R.

### RT‐qPCR Analysis

The meninges and spleens were collected post‐euthanasia of the mice. Total RNA from the meninges, spleen, brain, or cells was extracted using Trizol (AidLab) and reverse transcribed with the PrimeScript RT reagent Kit with gDNA Eraser (Takara). Quantitative PCR (qPCR) was conducted using primers spanning introns in the StepOnePlus Real‐Time PCR system (ThermoFisher).

The following primers were used:

*Ppib*, F‐TGGAGAGCACCAAGACAGACA, R‐TGCCGGAGTCGACAATGAT;

LCMV GP, F‐CATTCACCTGGACTTTGTCAGACTC, R‐GCAACTGCTGTGTTCCCGAAAC;

*Ifnγ*, F‐ATCTGGAGGAACTGGCAAAA, R‐TTCAAGACTTCAAAGAGTCTGAGGTA;

*Tnfα*, F‐CCAAGGCGCCACATCTCCCT, R‐GCTTTCTGTGCTCATGGTGT;

*Il1rl1*, F‐AGACCTGTTACCTGGGCAAG, R‐CACCTGTCTTCTGCTATTCTGGA;

*Il33*, F‐GGTGAACATGAGTCCCATCA, R‐CGTCACCCCTTTGAAGCTC.

*Il1β*, F‐AGTTGACGGACCCCAAAAG, R‐AGCTGGATGCTCTCATCAGG.

*Il2*, F‐GCTGTTGATGGACCTACAGGA, R‐TTCAATTCTGTGGCCTGCTT.

*Il6*, F‐GCTACCAAACTGGATATAATCAGGA, R‐CCAGGTAGCTATGGTACTCCAGAA.

*Ccl1*, F‐GGCTGCCGTGTGGATACAG, R‐AGGTGATTTTGAACCCACGTTT.

*Ccl2*, F‐CTCGGACTGTGATGCCTTAAT, R‐TGGATCCACACCTTGCATTTA.

*Ccl4*, F‐GCCCTCTCTCTCCTCTTGCT, R‐GGAGGGTCAGAGCCCATT.

*Ccl7*, F‐TTCTGTGCCTGCTGCTCATA, R‐TTGACATAGCAGCATGTGGAT.

*Cxcl1*, F‐GTGTCTAGTTGGTAGGGCATAAT, R‐CAGTCCTTTGAACGTCTCTGT.

*Actb*, F‐AGGGTGTGATGGTGGGAATG, R‐CCAGTTGGTAACAATGCCATGT.

*Cd80*, F‐ACCCCCAACATAACTGAGTCT, R‐TTCCAACCAAGAGAAGCGAGG.

*Cd86*, F‐TGTTTCCGTGGAGACGCAAG, R‐TTGAGCCTTTGTAAATGGGCA.

*Vcam1*, F‐AGTTGGGGATTCGGTTGTTCT, R‐CCCCTCATTCCTTACCACCC.

*Icam1*, F‐GTGATGCTCAGGTATCCATCCA, R‐CACAGTTCTCAAAGCACAGCG.

*Sele*, F‐ ATGCCTCGCGCTTTCTCTC, R‐GTAGTCCCGCTGACAGTATGC.

### The Culture and Treatment of Primary Meningeal Stromal Cells

After euthanizing the mice, the entire meninges were harvested and subsequently digested using 1 mg mL^−1^ collagenase D (Roche, 11088866001) and 50 µg mL^−1^ DNase I (Roche, 10104159001). Following termination of digestion, the mixture was filtered using a 100 µm strainer. Magnetic beads for CD45 (Biolegend, 480028) staining were applied, and negative selection was employed to remove immune cells. Subsequently, after lysing erythrocytes with ACK, the stromal cells were cultured in DMEM medium (Corning, 10‐013‐CVRC) supplemented with 10% FBS and PS.

Four days later, LCMV Armstrong was used to infect at an MOI ratio of 2:1, and fixation was performed using 1% PFA after the corresponding infection period. For calcium inhibition, 100 µM 2‐APB was added for 1 h before infection. For calcium activation, stromal cells were stimulated with 1 µg mL^−1^ ionomycin and 20 ng mL^−1^ PMA. Subsequently, staining procedures for mouse meningeal samples were followed. Imaging was conducted using a Nikon microscope, and ImageJ was utilized for subsequent data processing, including counting or statistical analysis.

### The Culture and Treatment of Primary Mast Cells

After euthanizing the mice, the large and small leg bones were collected, and the bone marrow was cultured in IMDM medium (Corning, 10‐016‐CV) supplemented with 10 ng mL^−1^ IL‐3 (Sino Biological, 51066‐MNAH), 30 ng mL^−1^ SCF (Sino Biological, 50487‐M08B), 10% FBS, and Penicillin‐Streptomycin. The medium was refreshed every 3–4 days, and flow cytometry was utilized to assess the maturation rate of BMMCs after 1 month (>98%).

Additionally, the peritoneal lavage fluid was collected after euthanasia and cultured in RPMI 1640 medium (Sigma, R8758) supplemented with 10 ng mL^−1^ IL‐3, 30 ng mL^−1^ SCF, 10% FBS, Penicillin‐Streptomycin. The medium was changed every 3–4 days, and after 10 days, flow cytometry was employed to determine the maturation rate (>98%) of PCMCs.

For inducible gene knockout or knock‐in mediated by CreERT2, the cells were treated with medium containing 1 µM 4‐OH tamoxifen for 2 days, followed by switching to medium without 4‐OH tamoxifen. After 4 days, transcriptome sequencing, transcription level analysis, protein level assays, and other tests were performed.

PCMCs were stimulated with 0.02, 0.2, 2, 20, 200 ng mL^−1^ IL‐33 for 4 h to perform transcription analysis (RT‐qPCR). PCMCs were primed with 20 or 200 ng mL^−1^ IL‐33 for 24 h, followed by 1 mM, 100 µM ATP, 10 µM ATP or 50 µg mL^−1^ compound 48/80 stimulation for 4 h to perform transcription analysis (RNA‐seq and RT‐qPCR), for 30 min to perform degranulation analysis (β‐hexosaminidase release assay and transmission electron microscopy), for 8 h to perform the analysis of cultured supernatant (ELISA), for real‐time to perform the analysis of live cell imaging. PCMCs were primed with 200 ng mL^−1^ IL‐33 for 24 h to perform chromatin accessibility analysis (ATAC‐seq). Similarly, PCMCs were primed with 200 ng mL^−1^ IL‐33 for 24 h, followed by 0.01, 0.1, 1, 10, 100 nm clozapine stimulation for various measurements.

For optogenetic cell experiments, light stimulation in alternating periods of 2 s of illumination with 25 ms pulses at 20 Hz for 4 h to perform transcription analysis (RT‐qPCR), for 30 min to perform degranulation analysis (β‐hexosaminidase release assay) was performed.

BMMCs were primed with 200 ng mL^−1^ IL‐33 for 24 h, followed by 1 mM, 100 µM ATP or 1 µg mL^−1^ ionomycin and 20 ng mL^−1^ PMA stimulation for 4 h to perform transcription analysis (RNA‐seq and RT‐qPCR). The same treatment was performed for 30 min to perform degranulation analysis (β‐hexosaminidase release assay and transmission electron microscopy), for 8 h to perform the analysis of cultured supernatant (ELISA).

### The Culture and Treatment of Primary Dendritic Cells

After euthanizing the mice, the femurs were collected, and the bone marrow was cultured in RPMI 1640 medium supplemented with 10 ng mL^−1^ IL‐4 (Sino Biological, 51084‐MNAE), 20 ng mL^−1^ GM‐SCF (Sino Biological, 51048‐MNAH), 10% FBS, and Penicillin‐Streptomycin. Non‐adherent BMDCs were harvested on day 7 of differentiation and subsequently utilized for experimental analyses.

PCMCs were primed with 200 ng mL^−1^ IL‐33 for 24 h, followed by stimulation with 1 mM ATP for 8 h to collect the culture supernatants. Subsequently, BMDCs were stimulated with the conditioned supernatants at a 1:1 volume ratio, and total RNA was extracted 4 h later for RT‐qPCR analysis.

### The Culture and Treatment of Endothelial Cells

The MAEC cell line (Beijing Beina Chuanglian Biotech Institute, BNCC359881) was cultured in DMEM supplemented with 10% FBS and Penicillin‐Streptomycin. PCMCs were primed with 200 ng mL^−1^ IL‐33 for 24 h, followed by stimulation with 1 mM ATP for 8 h to collect the culture supernatants. Subsequently, MAECs were treated with the conditioned supernatants at a 1:1 volume ratio, and total RNA was isolated 4 h later for RT‐qPCR analysis.

### RNA Sequencing and Analysis

Total RNA from the cells was extracted using Trizol (AidLab) following the chloroform/isoamyl alcohol method. mRNA enrichment and fragment were followed using oligo(dT) magnetic beads and a disruption reagent. The synthesis of cDNA, the repair of terminal, and the ligation of adapter were performed. DNBs and cPAS were used for SE50 or PE150 sequencing. The subsequent data analysis is the same to SMART‐seq except for differential gene detection with the only standard of adjusted *p* value < 0.05. In addition, we used the average expression TPM to calculate synergy for differently expressed genes. Synergy of gene = (TPM_IL‐33_Prime_ATP – TPM_No_Prime_NC)/((TPM_IL‐33_Prime_NC + TPM_No_Prime_ATP) – 2 x (TPM_No_Prime_NC)). If the synergy value of a gene is greater than 1, it is judged to be synergistical, otherwise it is dominated by IL‐33 or ATP alone. The same calculation method was used to calculate the synergy of IL‐33 and clozapine. The heatmap was completed by pheatmap (v1.0.12) in R.

### ELISA Measurement

The supernatant of the stimulated culture medium was harvested and measured following the instructions provided in the IL‐1β (Invitrogen, 88‐7013‐88), IL‐6 (BioLegend, 431304), TNF‐α (Biolegend, 430904) ELISA kits.

### Degranulation Assay

Degranulation was conducted following established protocols as described previously.^[^ ^96^ ^]^ Prior to the degranulation assay, cells were transferred into HEPES degranulation solution. Following a 30‐min incubation with the stimulation, the reaction was halted by centrifugation at 4 °C. The supernatant was then added to the p‐NAG (Yuanye, S10146) substrate. Cells were lysed using Triton X‐100, and the lysate was added to the p‐NAG substrate. The reaction was allowed for 2 h before stopping with glycine.

### Imaging with Transmission Electron Microscopy

The cells were initially fixed in a solution comprising 2% paraformaldehyde and 2.5% glutaraldehyde for 1 h, followed by four washes with PB buffer (0.1 m). Subsequently, the cells underwent post‐fixation with 1% osmium tetroxide and 1.5% tetrapotassium hexacyanoferrate trihydrate for 1 h at 23 °C. Afterward, ethanol dehydration was performed using graded solutions (50%, 70%, 80%, 90%, 100%, 100%, 100%, 100%) for 5 min each. This was followed by immersion in 1,2‐epoxypropane twice for 5 min each and gradual infiltration with a mixture of 1,2‐epoxypropane and Epon 812 resin for 8 h (SPI). Subsequent steps involved two immersions in pure Epon 812 resin, followed by polymerization in an oven at 60 °C. Blocks of polymerized resin were sectioned using a Leica EM UC7 ultramicrotome (Wetzlar). Ultra‐thin sections (80 nm) were mounted and dried on coated copper grids, followed by staining on‐grid with 2% uranyl acetate for 20 min and lead citrate for 5 min. Imaging was conducted using an H‐7650B transmission electron microscope (Hitachi).

### Live cell Imaging

WGA‐647 labeling was applied 30 min prior to imaging. Stimulation was introduced following 5 min of real‐time fluorescence imaging and recorded for an additional 10 min. For calcium imaging, stimulation was administered after 1 min of real‐time fluorescence imaging, and the calcium flow response was monitored until completion.

PCMCs were stimulated with conditioned medium from virus‐infected stromal cells (supernatant of stromal cells:mast cells = 1:10). BMMCs were stimulated with 200 ng mL^−1^ IL‐33, 200 nM ATP, or 1 µg mL^−1^ ionomycin + 20 nM PMA. Meninges was stimulated with 80 µM ATP *ex vivo*. Imaging of the whole tissue was carried out using a Nikon A1R HD25 or Olympus SpinSR10 confocal microscope, and subsequent data processing, including counting or statistics, was performed using NIS‐Element or Imaris.

### Metabolic Flux Assays

Bioenergetics in mast cells were measured according to previous methods.^[^ ^97^ ^]^ Before the experiment, Cell‐Tak (Corning, 354240) coated culture plates and rehydrated probe plates were prepared (Agilent). PCMCs or BMMCs were primed with 200 ng mL^−1^ IL‐33 for 24 h. Mast cells were diluted to the appropriate concentration (1 × 10^5^ cells per well of XF96 well plate) and seeded onto the pre‐coated culture plates. Cells were centrifuged at 1200 rpm for 5 min at room temperature to facilitate cell adhesion, and then incubated in a CO_2_‐free 37 °C incubator for 1 h. For OCR measurement, drug stimulation was as follows: injection 1, medium or 200 ng mL^−1^ IL‐33 or 1 mM ATP; injection 2, 2 µM oligomycin; injection 3, 1 µM FCCP; injection 4, 1 µM antimycin A and 1 µM rotenone. For ECAR measurement, drug stimulation was as follows: injection 1, medium or 200 ng mL^−1^ IL‐33 or 1 mM ATP; injection 2, 25 mM glucose; injection 3, 2 µM oligomycin; injection 4, 45 mM 2‐DG. Data were normalized to total protein determined by BCA assay.

### ATAC Sequencing and Analysis

50 000 live PCMCs were lysed and processed to isolate nuclei, followed by transposition with Tn5 transposase at 37 °C for 30 min.^[^ ^98^ ^]^ Equimolar adaptors were ligated, and libraries were amplified by PCR using the ATAC‐seq_Novogene Kit (Novogene). Libraries were purified with AMPure beads and quantified using Qubit. Sequencing was performed on the Illumina NovaSeq platform generating 150 bp paired‐end reads.

Raw reads were quality‐filtered using fastp (v0.20.0) to remove adaptor sequences, low‐quality reads. Clean reads were aligned to the reference genome using BWA (v0.7.12), and reads mapping to mitochondrial or chloroplast DNA were discarded. Only uniquely mapped, properly paired reads with MAPQ ≥13, were retained for downstream analysis.

Peak calling was performed using MACS2 (v2.2.7.1). Peaks were normalized to a fixed width of 500 bp centered on the summit. Motif analysis was conducted using HOMER (v4.11). Genomic annotation and nearest gene identification were performed using ChIPseeker. Differentially accessible peaks between groups were identified by merging peaks with bedtools and comparing mean RPM values.

### Quantification and Statistical Analysis

NIS‐Element and Imaris 9.7.2 (https://imaris.oxinst.com) was used to reconstruct the image stacks obtained from the volume imaging. Fluorescent images were analyzed by NIS‐Element, Imaris 9.7.2 or ImageJ2 2.3.0. The FACS data were gating with FlowJo 10.8.1. The data were analyzed with Graphpad Prism 9 (https://www.graphpad.com/scientific‐software/prism). For normalization, the viral titers obtained from wild‐type mice were set to one. For the comparisons of the two groups, statistical analyses were performed using unpaired two‐tailed Student's *t*‐test. Welch's correction was used when the variances of the samples were unequal. Mann‐Whitney tests was used when the samples did not conform to a Gaussian distribution. Additionally, for statistical analyses of the single‐cell RNA sequencing data, the default Mann‐Whitney U test in **Seurat** was used. For RNA sequencing data, the default **Wald test** in **DESeq2** was applied. The sample size can be found in the figure legends. Each n represents the number of mice, cells, cell wells or images and is indicated in the figure legends. *p* ≤ 0.05 was considered significant. No statistical methods were used to predetermine sample size. Error bars represent SEM.

## Conflict of Interest

The authors declare no conflict of interest.

## Author Contributions

Q.L., W.C., and M.S. contributed equally to this work. Q.L. and W.C. performed and analyzed experiments; M.S. performed the majority of the flow cytometry analyses on spleen and peripheral blood samples; X.N. contributed to the viral propagation procedures; X.H. participated in the project discussions and provided valuable insights; Q.R. and F.C. provided the human meninges sample; W.Z. directed the project, conceived the experiments, and provided supervision. The manuscript was written by W.Z., with assistance from Q.L.

## Supporting information



Supporting Information

Supplemental Video 1

## Data Availability

The data that support the findings of this study are available from the corresponding author upon reasonable request. The raw and processed data of scRNA‐seq, SMART‐seq, bulk RNA‐seq and ATAC‐seq are deposited in the National Center for Biotechnology Information's Gene Expression Omnibus as SuperSeries under accession numbers GSE263671.
