# Supplementary material for: Stromal Cell‐Mast Cell Communication Orchestrates Anti‐Viral Immunity in the Meninges
Source: Adv Sci (Weinh). 2025 Nov 6;13(5):e14842. doi: 10.1002/advs.202514842 (PMC12850450; doi:10.1002/advs.202514842)
Supplement: Supplementary file 1 — Supporting Information [file ADVS-13-e14842-s002.docx]

Supporting Information

Stromal Cell-Mast Cell Communication Orchestrates Anti-Viral Immunity in the Meninges

Qingqing Li, Weijia Chen, Mengxue Sun, Xinbo Ni, Qishan Ran, Xiaoyu Hu, Fang Cao*, Wenwen Zeng*

**Figure S1. *Tpsb2-CreERT2* enables efficient and specific labeling of mast cells, revealing their meningeal distribution. Related to Figure 1.**

**
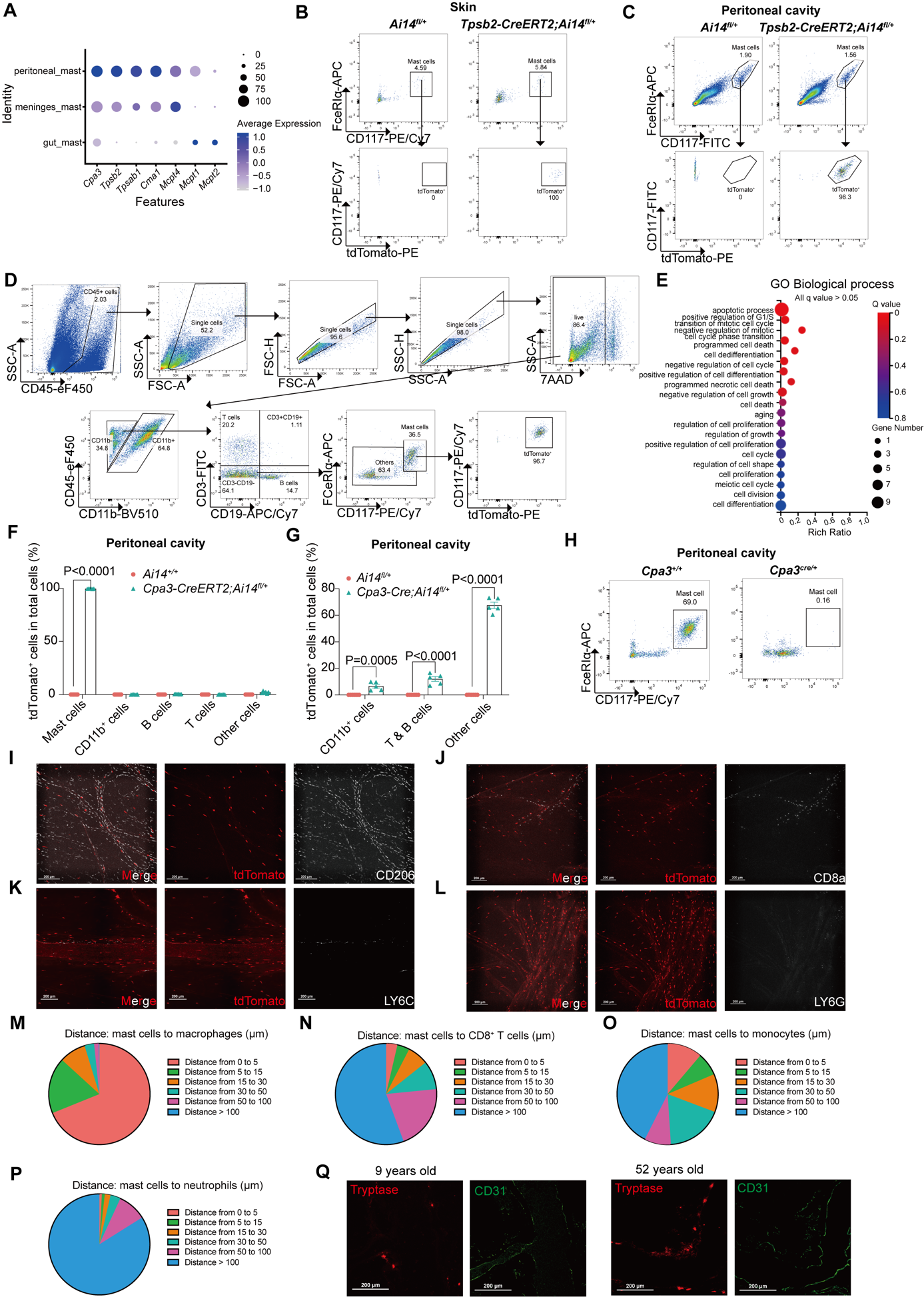
**

A) Mast cell marker gene dot plot from various tissues. B) Representative contour plot of dorsal skin CD45^+^CD11b^-^CD3e^-^CD19^-^ cells from *Tpsb2-CreERT2; Ai14^fl/+^* mice. n=2 (mice). C) Representative contour plot of peritoneal CD45^+^ cells from *Tpsb2-CreERT2; Ai14^fl/+^* mice. n=11 (mice). D) Gating strategy for meningeal mast cells and other cells. E) The GO biological process pathway enrichment analysis in peritoneal mast cells from *Tpsb2-CreERT2; Ai14^fl/+^* mice. n=3 (mice). F) Quantification of labeled mast cells in peritoneal immune cells from *Cpa3-CreERT2; Ai14^fl/+^* mice. n=6 (mice). G) Quantification of labeled immune cell subsets in peritoneal immune cells from *Cpa3-Cre; Ai14^fl/+^* mice. n=5-6 (mice). H) Representative contour plot of peritoneal CD45^+^CD11b^-^CD3e^-^CD19^-^ cells from *Cpa3^cre/+^* mice. n=5-6 (mice). I-P) Maximal intensity projection of dural meninges stained for mast cells (red) and various markers (white) from *Tpsb2-CreERT2; Ai14^fl/+^* mice. I) Macrophages (white). n=3 (mice). J) CD8⁺ T cells (white). n=3 (mice). K) Monocytes (white). n=3 (mice). L) Neutrophils (white). n=3 (mice). M) Quantification of (I). N) Quantification of (J). O) Quantification of (K). P) Quantification of (L). Q) Staining of mast cells (red) and blood vessel (green) in meninges from patient 1 (left, 9 years old female) and 2 (right, 52 years old female). Scale bar for all images, 200 μm. Data are presented as mean ± SEM and *p* values were calculated by Wald test from DESeq2 (E), two-tailed, unpaired Student’s t-test (F-G).

**Figure S2. Meningeal mast cells mount a potent immune response to viral infection. Related to Figure 2.**

**
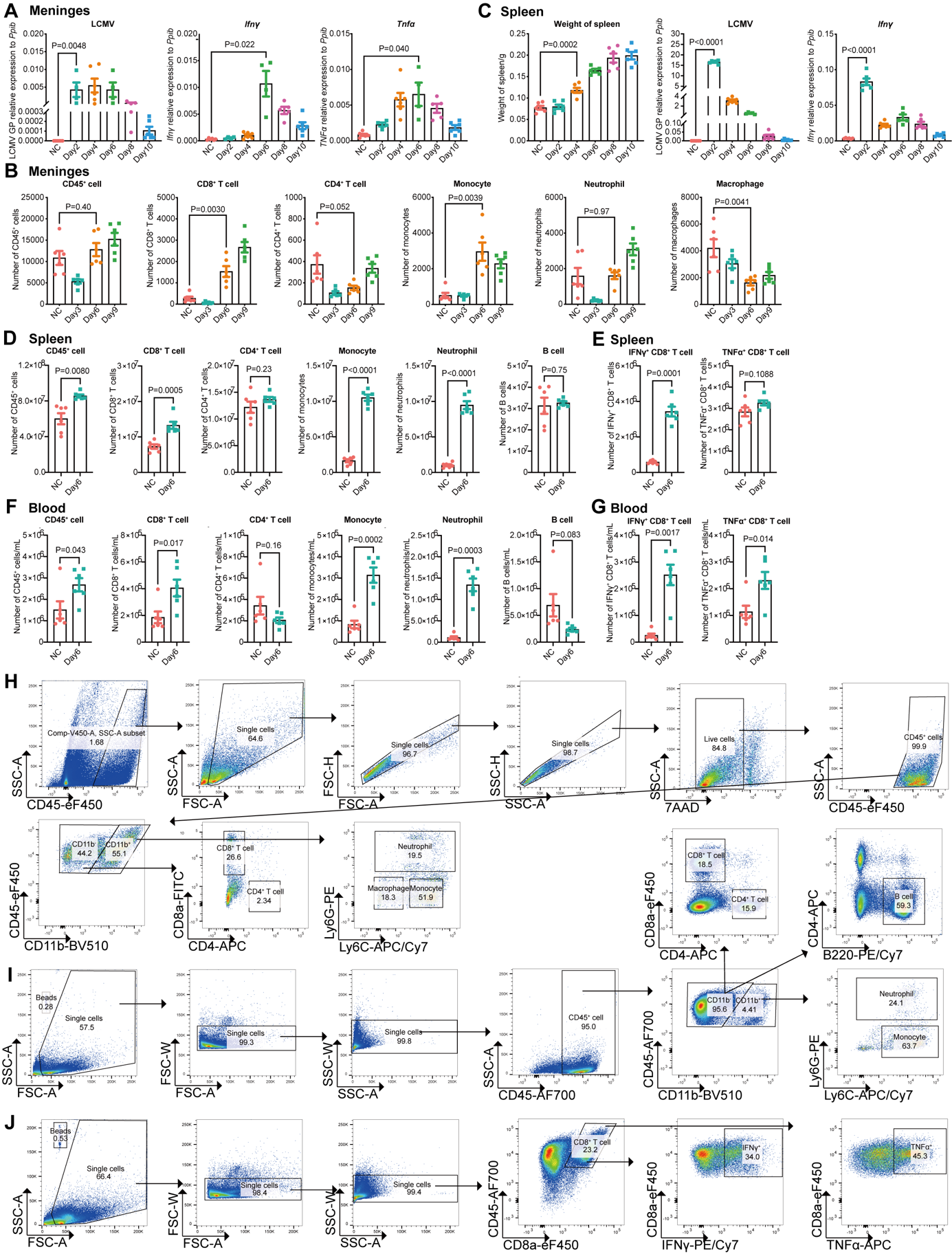
**

A) LCMV, *Ifnγ*, and *Tnfα* mRNA levels in the dural meninges at day 0, 2, 4, 6, 8, and 10 post LCMV infection. n=4-6 (mice). B) Meningeal cell counts at day 0, 3, 6, and 9 post LCMV infection. n=6 (mice). C) The weight and LCMV, *Ifnγ* mRNA levels in the spleen at day 0, 2, 4, 6, 8, and 10 post LCMV infection. n=6 (mice). D) Spleen cell counts at day 0 and 6 post LCMV infection. n=6 (mice). E) The IFN-γ and TNF-α production by spleen CD8⁺ T cells from at day 0 and 6 post LCMV infection. n=6 (mice). F) Peripheral blood cell counts at day 0 and 6 post LCMV infection. n=6 (mice). G) The IFN-γ and TNF-α production by peripheral blood CD8⁺ T cells from at day 0 and 6 post LCMV infection. n=6 (mice). H) Gating strategy for the meninges. I) Gating strategy for peripheral blood and spleen. J) Gating strategy for IFN-γ and TNF-α production by CD8^+^ T cells *in vitro*. Data are presented as mean ± SEM and *p* values were calculated by two-tailed, unpaired Student’s t-test (A-G).

**Figure S3. Meningeal mast cells mount a potent immune response to viral infection. Related to Figure 2.**

**
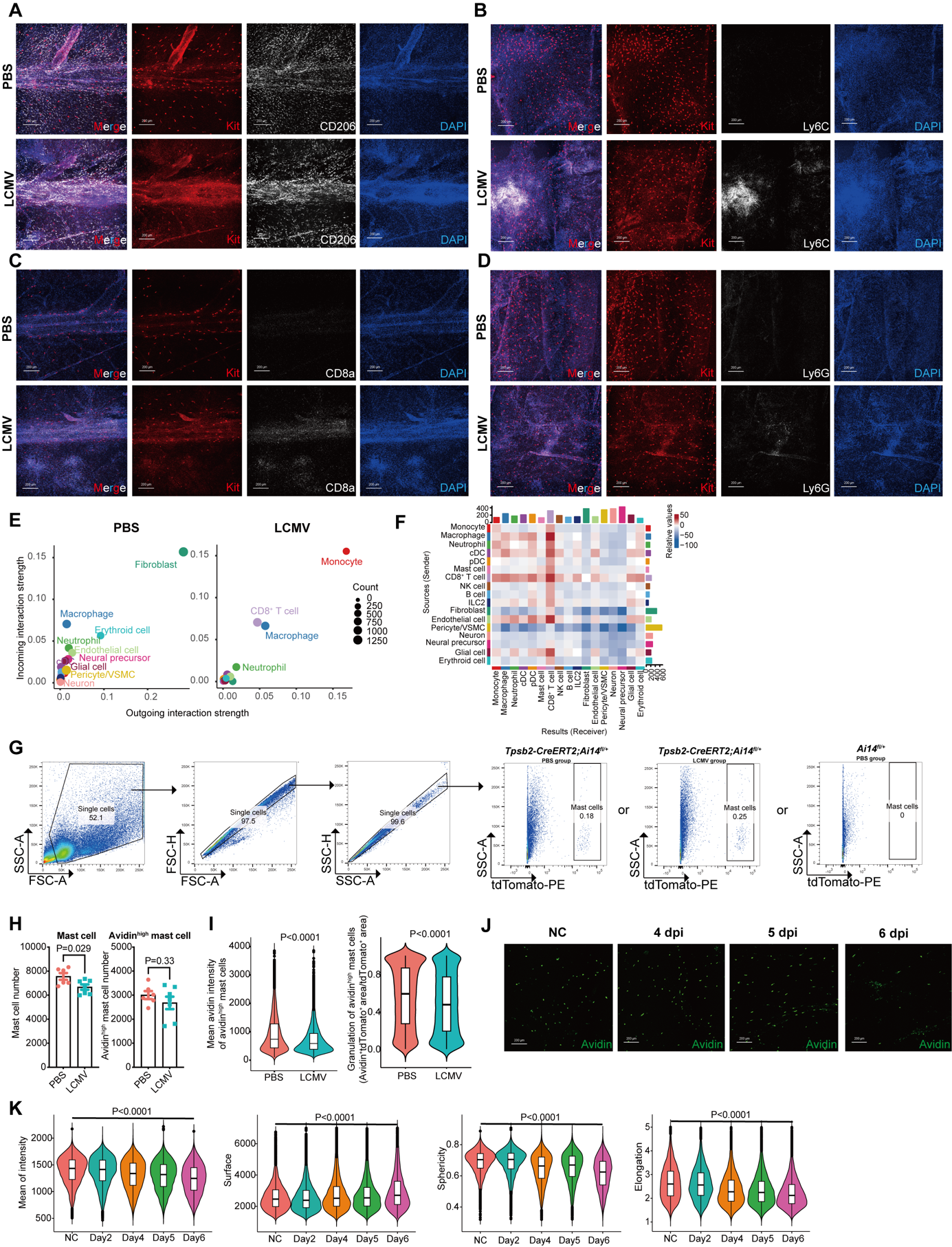
**

A-D) Maximal intensity projection of dural meninges stained for mast cell (red), various markers (white), and nuclei (blue) from un-infected (PBS) and infected (LCMV) mice at 6 dpi. A) Macrophage (white). B) Monocyte (white). C) CD8^+^ T cell (white). n=3 (mice). D) Neutrophil (white). E) Incoming and outgoing interaction strength of meningeal sc-RNA seq at 6 dpi. F) Cell to cell commutations of meningeal sc-RNA seq at 6 dpi. G) Gating and sorting strategy of meningeal mast cells with or without 6 days of LCMV infection for SMART-seq. H) The number of meningeal mast cells (tdTomato^+^ cells) and meningeal avidin^high^ mast cells (avidin^high^tdTomato^+^ cells) at 6 dpi. n=6-7 (mice). I) Quantification of granule mean intensity and granulation of avidin^high^ mast cells at 6 dpi. n=11,504-12,795 (cells). J) Maximal intensity projection of dural meninges stained for granule (green) at 0, 4, 5, 6 dpi. n=3-4 (mice). K) Quantification of mean intensity, granule surface, sphericity and elongation of avidin^high^ mast cells (J). N = 7,291-9,216 (cells). Scale bar for all images, 200 μm. Data are presented as mean ± SEM and *p* values were calculated by two-tailed, unpaired Student’s t-test (H-I, K).

**Figure S4. Mast cell depletion inhibits immune cell recruitment to the meninges. Related to Figure 3.**

**
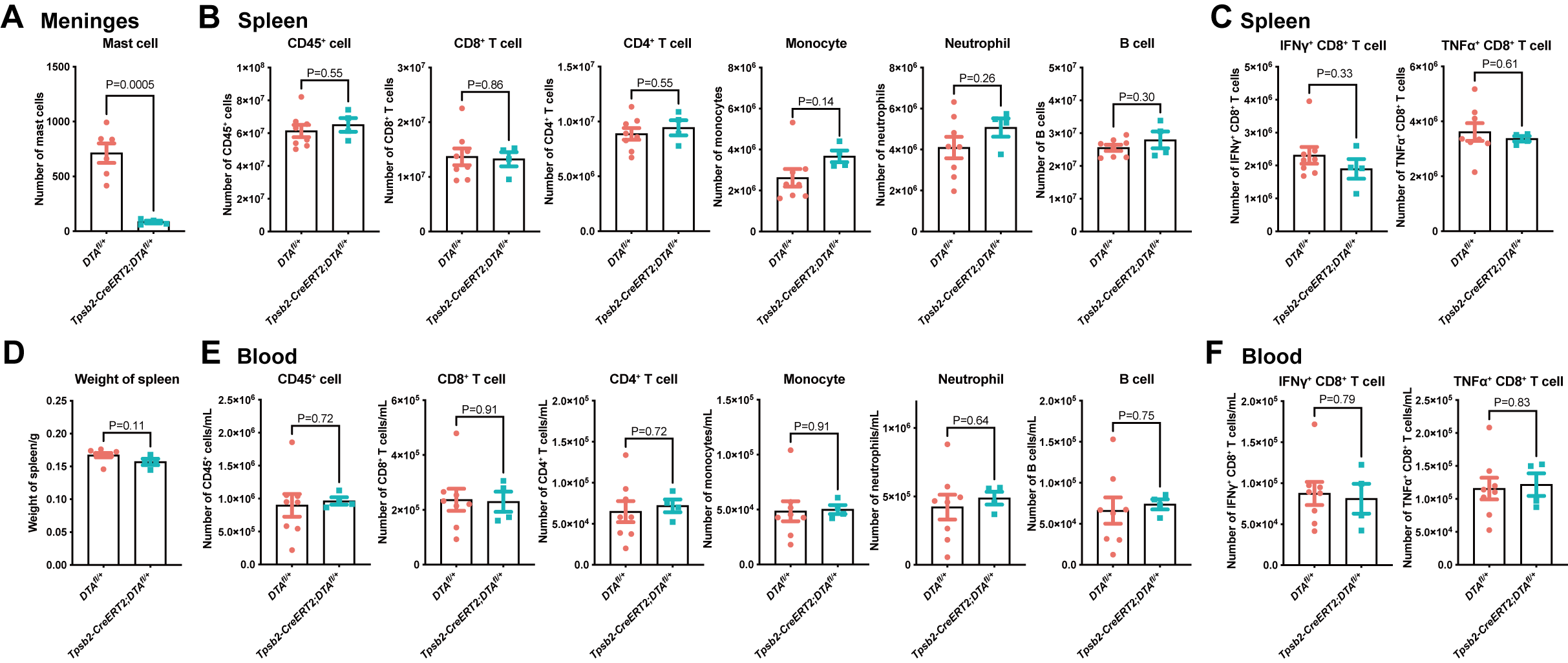
**

A) Meningeal mast cell counts from *Tpsb2-CreERT2; DTA^fl/+^* mice. n=4-6 (mice). B) Spleen cell counts from *Tpsb2-CreERT2; DTA^fl/+^* mice. n=4-8 (mice). C) The IFN-γ and TNF-α production of spleen CD8^+^ T⁺ cells from *Tpsb2-CreERT2; DTA^fl/+^* mice at 6 dpi. n=4-8 (mice). D) Weight of spleen from *Tpsb2-CreERT2; DTA^fl/+^* mice. n=4-8 (mice). E) Peripheral blood cell counts from *Tpsb2-CreERT2; DTA^fl/+^* mice. n=4-8 (mice). F) The IFN-γ and TNF-α production by peripheral blood CD8^+^ T cells from *Tpsb2-CreERT2; DTA^fl/+^* mice at 6 dpi. n=4-8 (mice). Data are presented as mean ± SEM and *p* values were calculated by two-tailed, unpaired Student’s t-test (A-F).

**Figure S5. Mast cell activation promotes immune cell recruitment to the meninges. Related to Figure 3.**

**
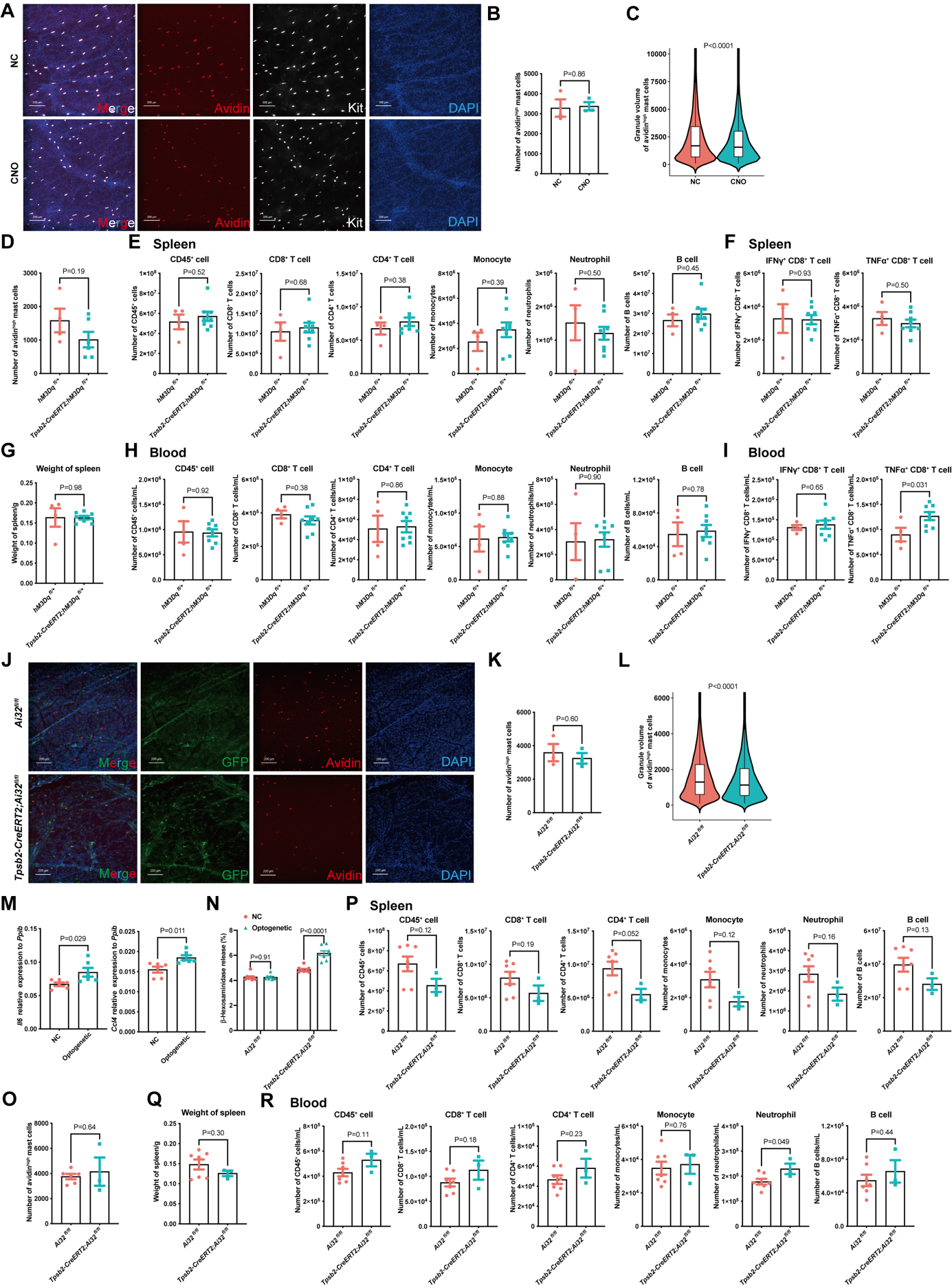
**

A-I) Pharmacogenetic activation of mast cells was employed with *Tpsb2-CreERT2; hM3Dq^fl/+^* mice. A) Maximal intensity projection of dural meninges stained for granule (red), mast cell (white), and nuclei (blue). n=3 (mice). B) Number of meningeal avidin^high^ mast cells (A). C) Granulated volume of avidin^high^ mast cells (A). n=9,847-10,110 (cells). D) Number of meningeal avidin^high^ mast cells at 5.6 dpi. n=4-6 (mice). E) Spleen cell counts at 5.6 dpi. n=4-6 (mice). F) The IFN-γ and TNF-α production by spleen CD8^+^ T cells at 5.6 dpi. n=4-6 (mice). G) Weight of spleen at 5.6 dpi. n=4-6 (mice). H) Peripheral blood counts at 5.6 dpi. n=4-6 (mice). I) The IFN-γ and TNF-α production by peripheral blood CD8^+^ T cells at 5.6 dpi. n=4-6 (mice). J-R) Optogenetic activation of mast cells was employed with *Tpsb2-CreERT2; Ai32^fl/fl^* mice. J) Maximal intensity projection of dural meninges stained for granule (red), mast cell (green), and nuclei (blue). n=3 (mice). K) Number of meningeal avidin^high^ mast cells (J). L) Granulated volume of avidin^high^ mast cells (J). n=9,744-10,767 (cells). M) PCMCs were stimulated with optogenetic blue light for 4 h followed by RT-qPCR. The relative transcript levels of *Il6* and *Ccl4* mRNA levels are shown. n=6 (wells). N) PCMCs were stimulated with optogenetic blue light for 30 min followed by measurement of β-hexosaminidase release assay. n=8 (wells). O) Number of meningeal avidin^high^ mast cells at 5.6 dpi. n=3-7 (mice). P) Spleen cell counts at 5.6 dpi. n=3-7 (mice). Q) Weight of spleen at 5.6 dpi. n=3-7 (mice). R) Peripheral blood counts at 5.6 dpi. n=3-7 (mice). Scale bar for all images, 200 μm. Data are presented as mean ± SEM and *p* values were calculated by two-tailed, unpaired Student’s t-test (B-I, K-R).

**Figure S6. ST2 signal on meningeal mast cells promotes the antiviral immune response and CD8^+^ T cell infiltration. Related to Figure 4.**

**
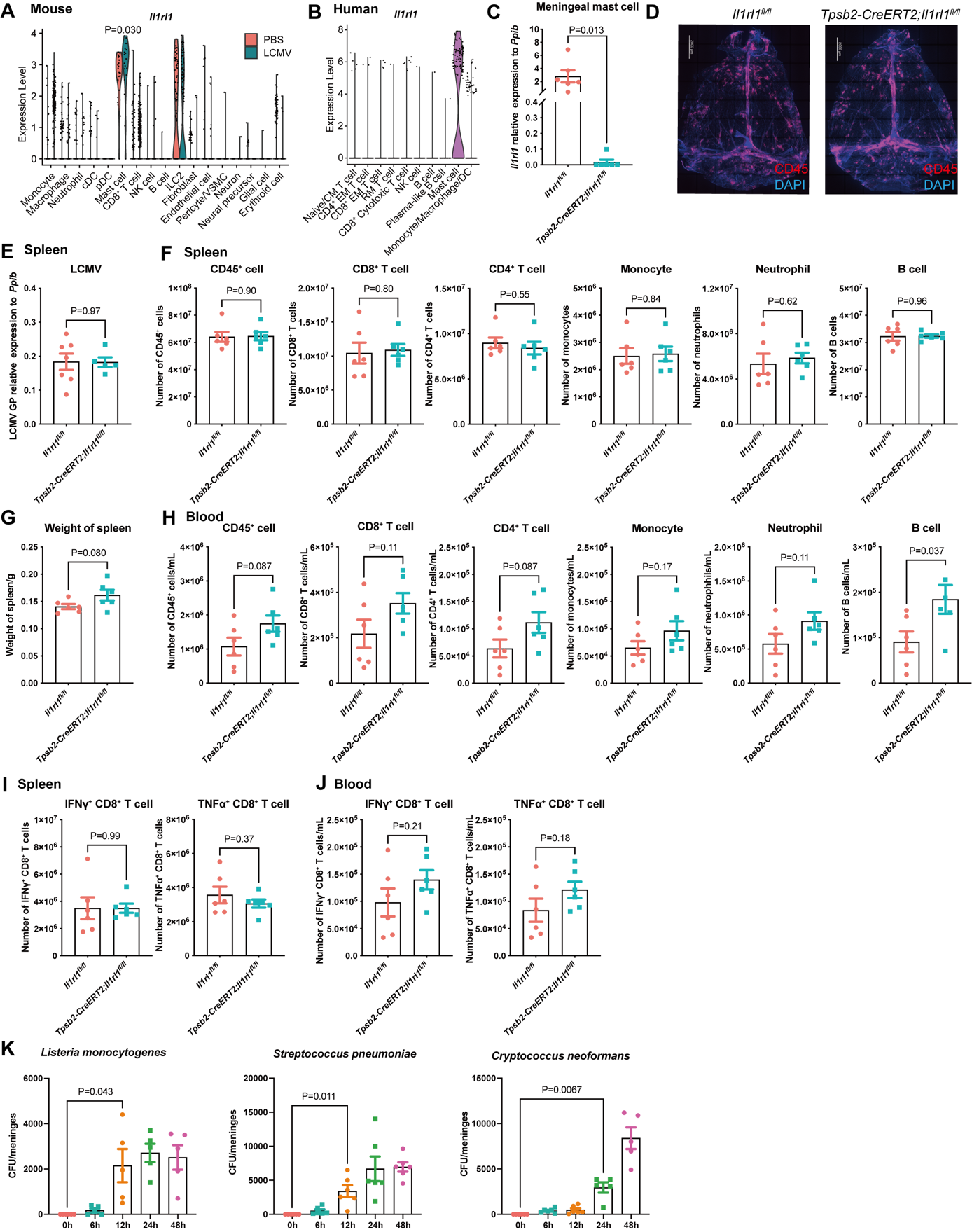
**

A) *Il1rl1* expression level (log_2_(CPM+1)) in the meninges of mice analyzed by sc-RNA seq. B) *Il1rl1* expression level (log_2_(CPM+1)) in the meninges of human analyzed by sc-RNA seq.

C-J) *Tpsb2-CreERT2; Il1rl1^fl/fl^* mice were used to delete ST2 in mast cell. C) *Il1rl1* mRNA levels in the meningeal mast cells. n=6 (mice). D) Maximal intensity projection of dural meninges stained for immune cells (red) and nuclei (blue) at 6 dpi. Scale bar, 2000 μm (whole mount). n=7-8 (mice). E) LCMV mRNA levels in the spleen at 6 dpi. n=5-7 (mice). F) Spleen cell counts at 6 dpi. n=6 (mice). G) Weight of spleen at 6 dpi. n=6 (mice). H) Peripheral blood cell counts at 6 dpi. n=6 (mice). I) The IFN-γ and TNF-α production by spleen CD8^+^ T cells at 6 dpi. n=6 (mice). J) The IFN-γ and TNF-α production by peripheral blood CD8^+^ T cells at 6 dpi. n=6 (mice). K) *Listeria monocytogenes*, *Streptococcus pneumoniae*, *Cryptococcus neoformans* burden levels in the dural meninges at 0, 6, 12, 24 and 48 hours post infection. n=5-6 (mice). Data are presented as mean ± SEM and *p* values were calculated by Mann-Whitney U test from Seurat (A), two-tailed, unpaired Student’s t-test (C, E-K).

**Figure S7. IL-33 signal from meningeal stromal cells promotes the antiviral immune response and CD8^+^ T cell infiltration. Related to Figure 5.**

**
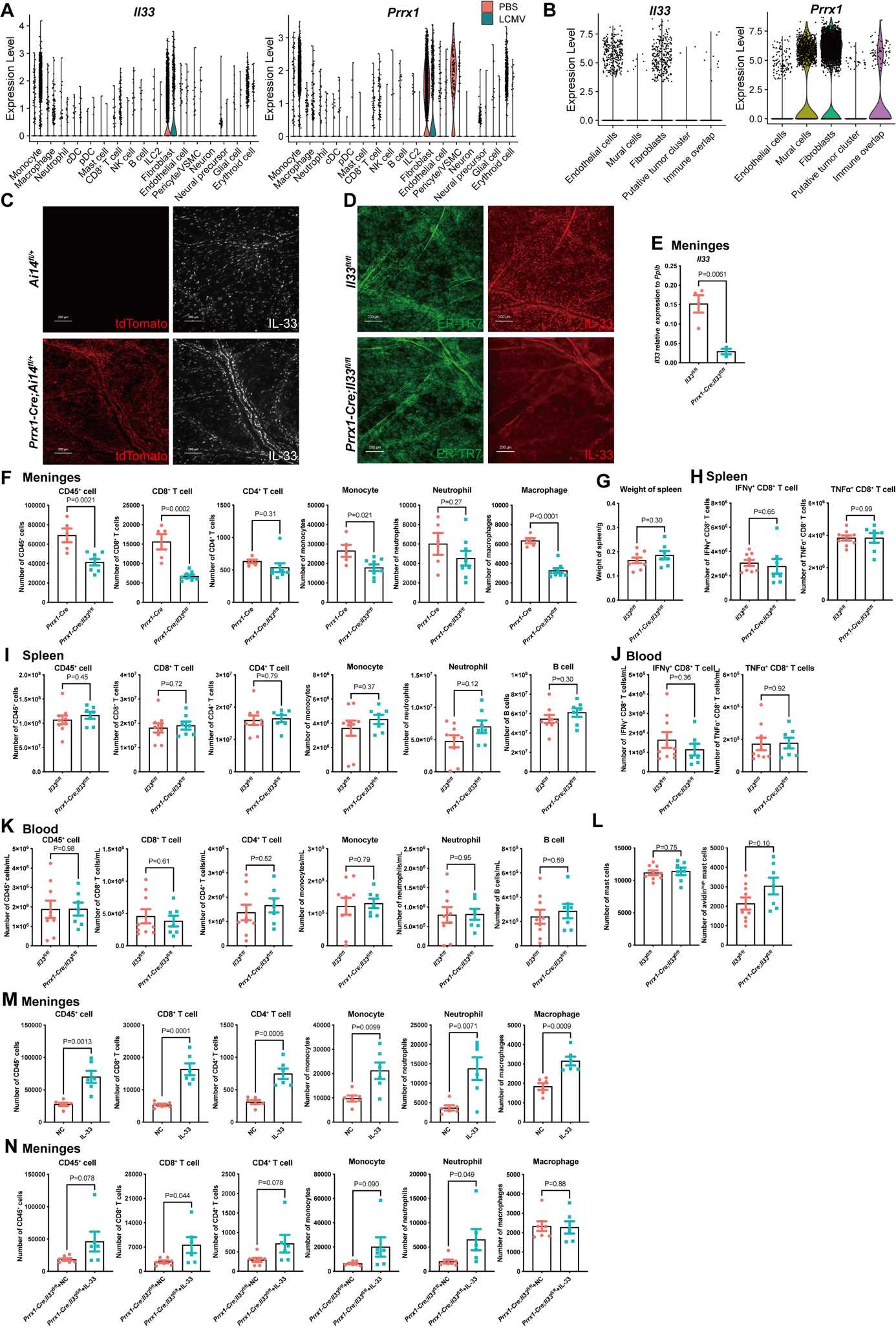
**

A) *Il33* and *Prrx1* expression levels (log_2_(CPM+1)) in the meninges of mice analyzed by sc-RNA seq. B) *Il33* and *Prrx1* expression levels (log_2_(CPM+1)) in the meninges of human analyzed by sc-RNA seq. C) Maximal intensity projection of dural meninges stained for stromal cell (red) and IL-33 (white) from *Prrx1-Cre; Ai14^fl/+^* mice. Scale bar, 200 μm. n=4-6 (mice). D-N) *Prrx1-Cre; Il33^fl/fl^* mice were used to delete IL-33 in stromal cell. D) Maximal intensity projection of dural meninges stained for stromal cell (green) and IL-33 (red). Scale bar, 200 μm. n=3 (mice). E) *Il33* mRNA levels in the dural meninges. n=3-4 (mice). F) Meningeal cell counts at 6 dpi. n=5-8 (mice). G) Weight of spleen at 6 dpi. n=7-9 (mice). H) The IFN-γ and TNF-α production by spleen CD8^+^ T cells at 6 dpi. n=7-9 (mice). I) Spleen cell counts at 6 dpi. n=7-9 (mice). J) The IFN-γ and TNF-α production by peripheral blood CD8^+^ T cells at 6 dpi. n=7-9 (mice). K) Peripheral blood counts at 6 dpi. n=7-9 (mice). L) Number of meningeal mast cells, avidin^high^ mast cells at 6 dpi. n=7-9 (mice). M) Meningeal cell counts from treated (mouse IL-33 recombinant protein) mice at 5.6 dpi. n=6 (mice). N) Meningeal cell counts from treated (mouse IL-33 recombinant protein) *Prrx1-Cre; Il33^fl/fl^* mice at 5.6 dpi. n=6-7 (mice). Data are presented as mean ± SEM and *p* values were calculated by two-tailed, unpaired Student’s t-test (E-N).

**Figure S8. Meningeal stromal cell-derived IL-33 and ATP coordinate antiviral defense Related to Figure 6.**

**
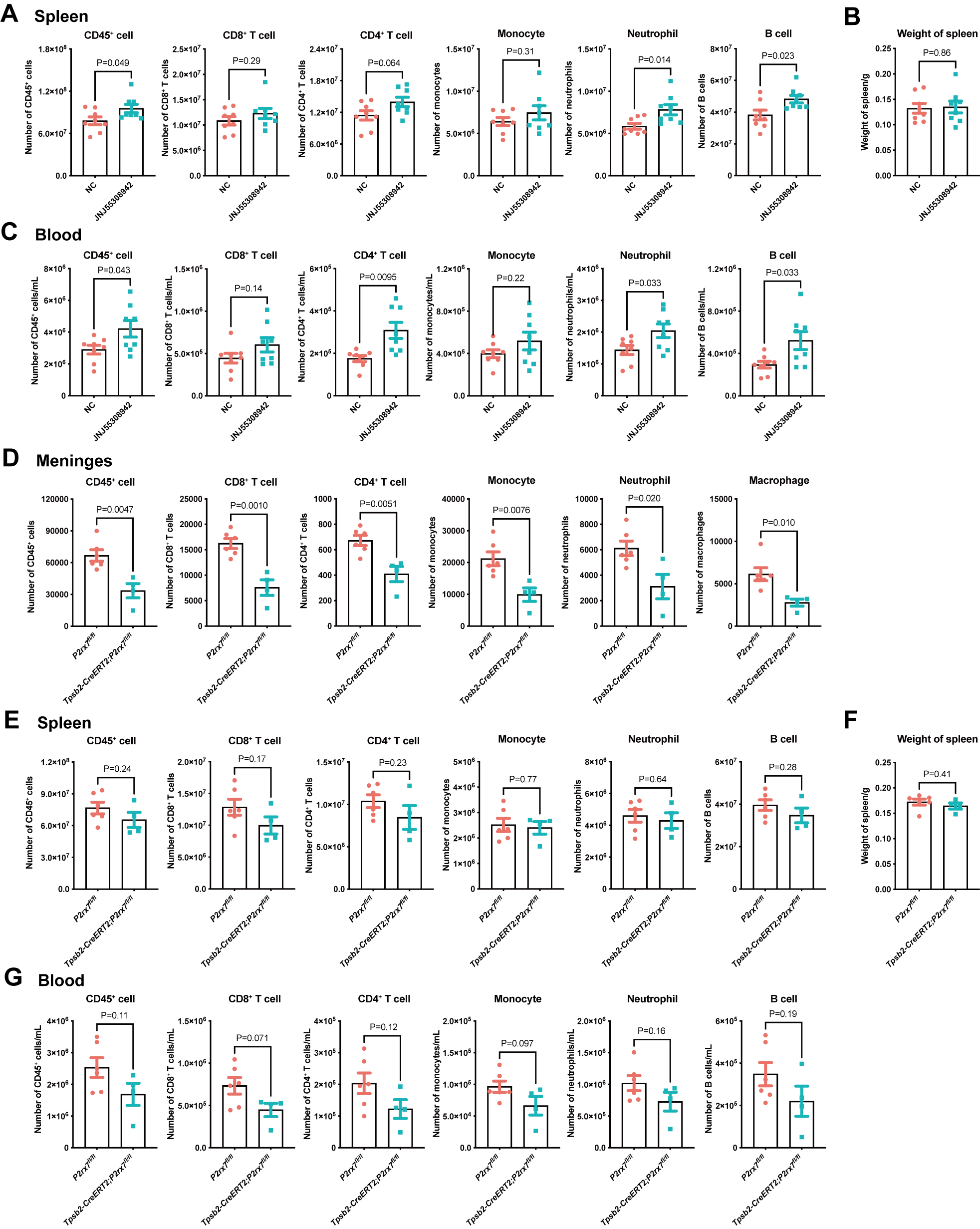
**

A) Spleen cell counts from ATP receptor antagonist (JNJ55308942) treated mice at 6 dpi. n=8 (mice). B) Weight of spleen from ATP receptor antagonist (JNJ55308942) treated mice at 6 dpi. n=8 (mice). C) Peripheral blood cell counts from ATP receptor antagonist (JNJ55308942) treated mice at 6 dpi. n=8 (mice). D-G) *Tpsb2-CreERT2; P2rx7^fl/fl^* mice were used to delete P2RX7 in mast cell. D) Meningeal cell counts at 6 dpi. n=4-6 (mice). E) Spleen cell counts at 6 dpi. n=4-6 (mice). F) Weight of spleen at 6 dpi. n=4-6 (mice). G) Peripheral blood cell counts at 6 dpi. n=4-6 (mice). Data are presented as mean ± SEM and *p* values were calculated by two-tailed, unpaired Student’s t-test (A-G).

**Figure S9. IL-33 licenses mast cells for potent activation by ATP. Related to Figure 7.**

**
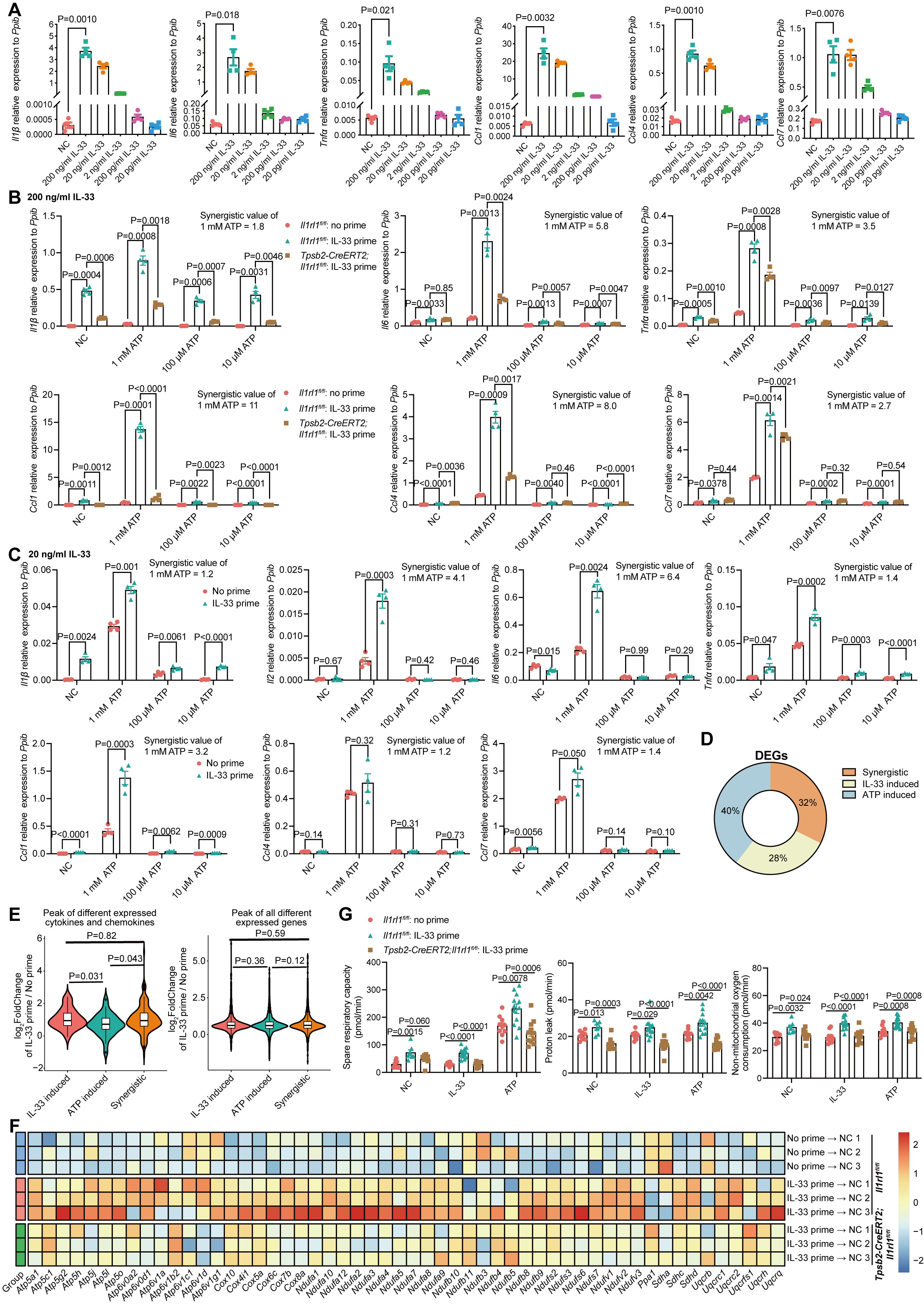
**

A) PCMCs were treated with 0.02, 0.2, 2, 20, 200 ng/ml IL-33 for 4 h followed by RT-qPCR. The mRNA levels of *Il1β, Il6, Tnfα, Ccl1, Ccl4,* and *Ccl7*. n=4 (wells). B-G) PCMCs from control (*Il1rl1^fl/fl^*) and ST2-deleted (*Tpsb2-CreERT2; Il1rl1^fl/fl^*) mice were treated with IL-33 for 24 h, then stimulated with ATP followed by RT-qPCR (B-C), RNA-seq (D, F), ATAC-seq (E), and seahorse (G). B) The mRNA levels of *Il1β, Il6, Tnfα, Ccl1, Ccl4,* and *Ccl7*. n=4 (wells). C) The mRNA levels of *Il1β, Il2, Il6, Tnfα, Ccl1, Ccl4,* and *Ccl7*. n=4 (wells). D) The synergy of differentially expressed genes. E) Peak RPM log_2_FoldChange of IL-33 induced, ATP induced and synergistic differentially expressed genes. F) Heatmap of other differentially expressed oxidative phosphorylation related genes. n=3 (wells). G) OCR measurement. n=6-12 (wells). Data are presented as mean ± SEM and *p* values were calculated by two-tailed, unpaired Student’s t-test (A-C, E, G), Wald test from DESeq2 (D, F).

**Figure S10. IL-33 licenses mast cells for potent activation by ATP. Related to Figure 7.**

**
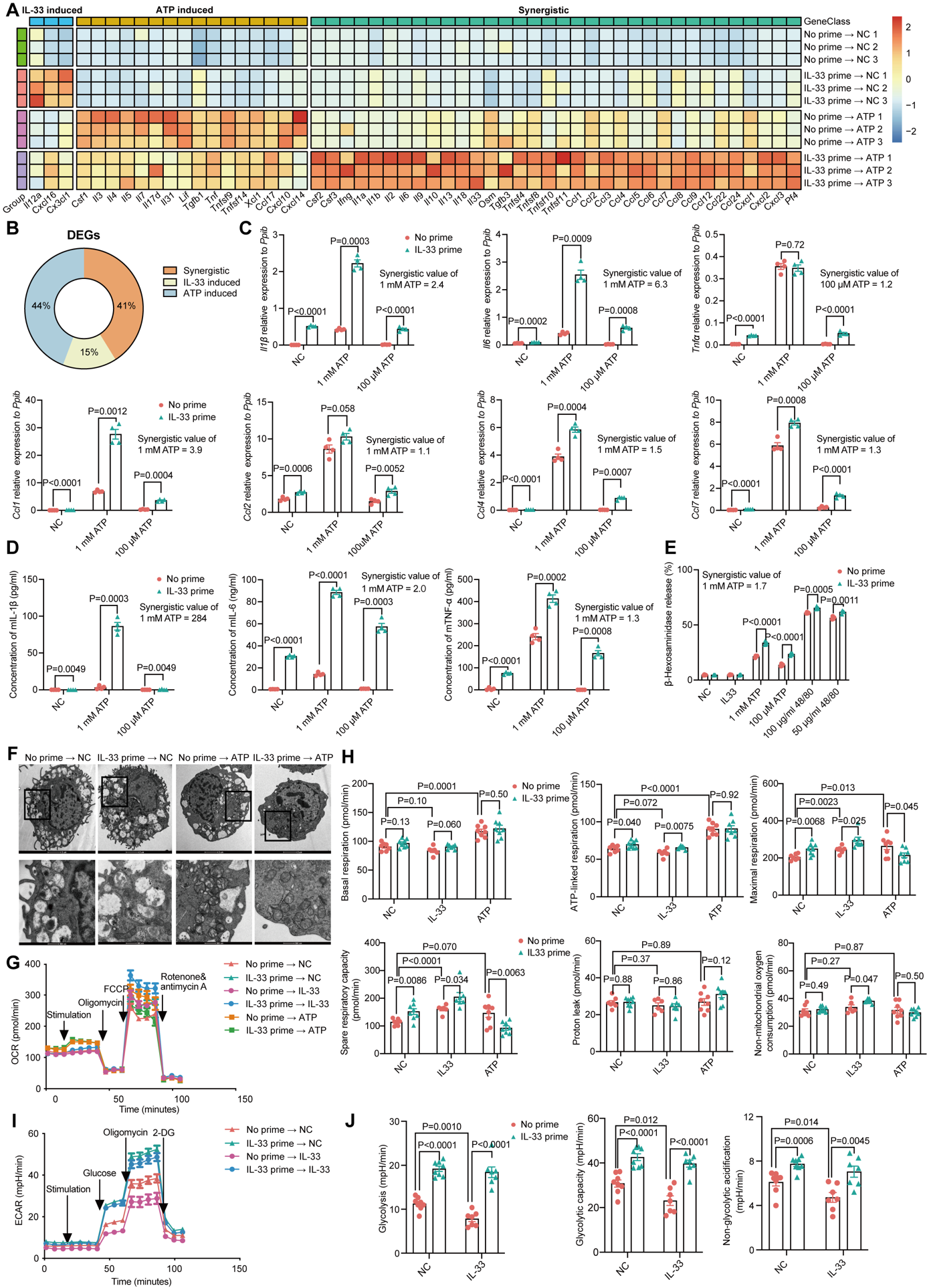
**

A-J) BMMCs were treated with IL-33 for 24 h, then stimulated with ATP followed by RNA seq (A-B), RT-qPCR (C), ELISA (D), degranulation (E-F), and seahorse (G-J). A) Heatmap of differentially expressed cytokines and chemokines. n=3 (wells). B) The synergy of differentially expressed genes. C) The mRNA levels of *Il1β, Il6, Tnfα, Ccl1, Ccl2, Ccl4,* and *Ccl7*. n=4 (wells). D) The protein levels of IL-1β, IL-6, and TNF-α. n=4 (wells). E) Degranulation measurement of β-hexosaminidase release assay. n=4 (wells). F) Degranulation imaging with transmission electron microscopy. Scale bar, 2 μm (top), 500 nm (bottom). G) OCR measurement. n=7-8 (wells). H) Quantification of (G). I) ECAR measurement. n=7-8 (wells). J) Quantification of (I). Data are presented as mean ± SEM and *p* values were calculated by Wald test from DESeq2 (A, B), two-tailed, unpaired Student’s t-test (C-E, H, J).

**Figure S11. IL-33 licenses mast cells for potent activation by ATP. Related to Figure 7.**

**
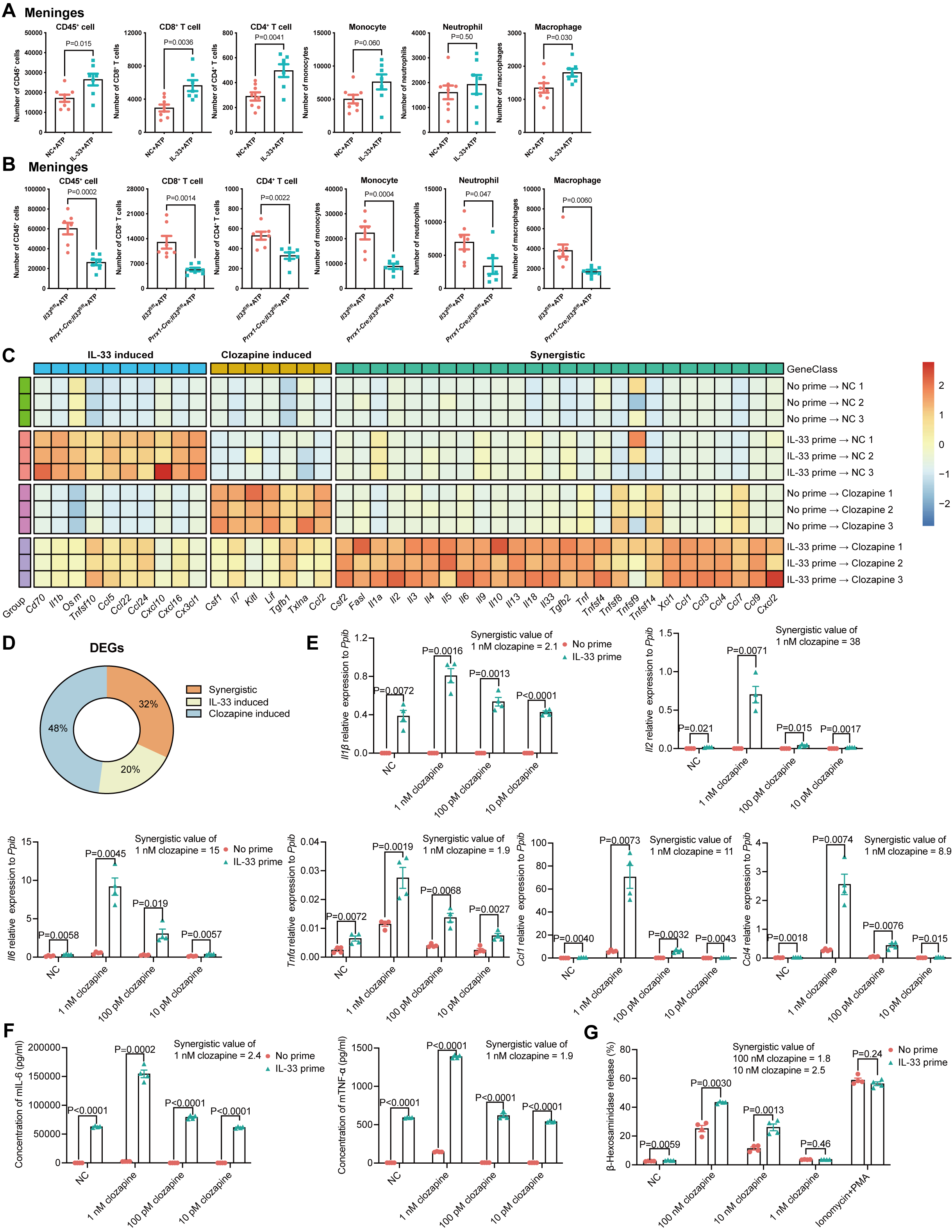
**

A) Meningeal cell counts from control (NC) and treated (mouse IL-33 recombinant protein) mice with the supplementation of ATP at 5.6 dpi. n=7-8 (mice). B) Meningeal cell counts from control (*Il33^fl/fl^*) and IL-33-deleted (*Prrx1-Cre; Il33^fl/fl^*) mice with the supplementaion of ATP at 6 dpi. n=7 (mice). C-G) PCMCs were obtained from *Tpsb2-CreERT2; hM3Dq^fl/+^* mice and treated with IL-33 for 24 h, then stimulated with clozapine followed by RNA seq (C-D), RT-qPCR (E), ELISA (F), and degranulation (G). C) Heatmap of differentially expressed cytokines and chemokines. n=3 (wells). D) The synergy of differentially expressed genes. E) The mRNA levels of *Il1β, Il2, Il6, Tnfα, Ccl1* and *Ccl4*. n=4 (wells). F) The protein levels of IL-6 and TNF-α. n=4 (wells). G) Degranulation measurement of β-hexosaminidase release assay. n=4 (wells). Data are presented as mean ± SEM and *p* values were calculated by two-tailed, unpaired Student’s t-test (A-B, E-G), Wald test from DESeq2 (C, D).

**Figure S12. Mast cells promote CD8⁺ T cell infiltration through direct and indirect mechanisms. Related to Figure 7.**

**
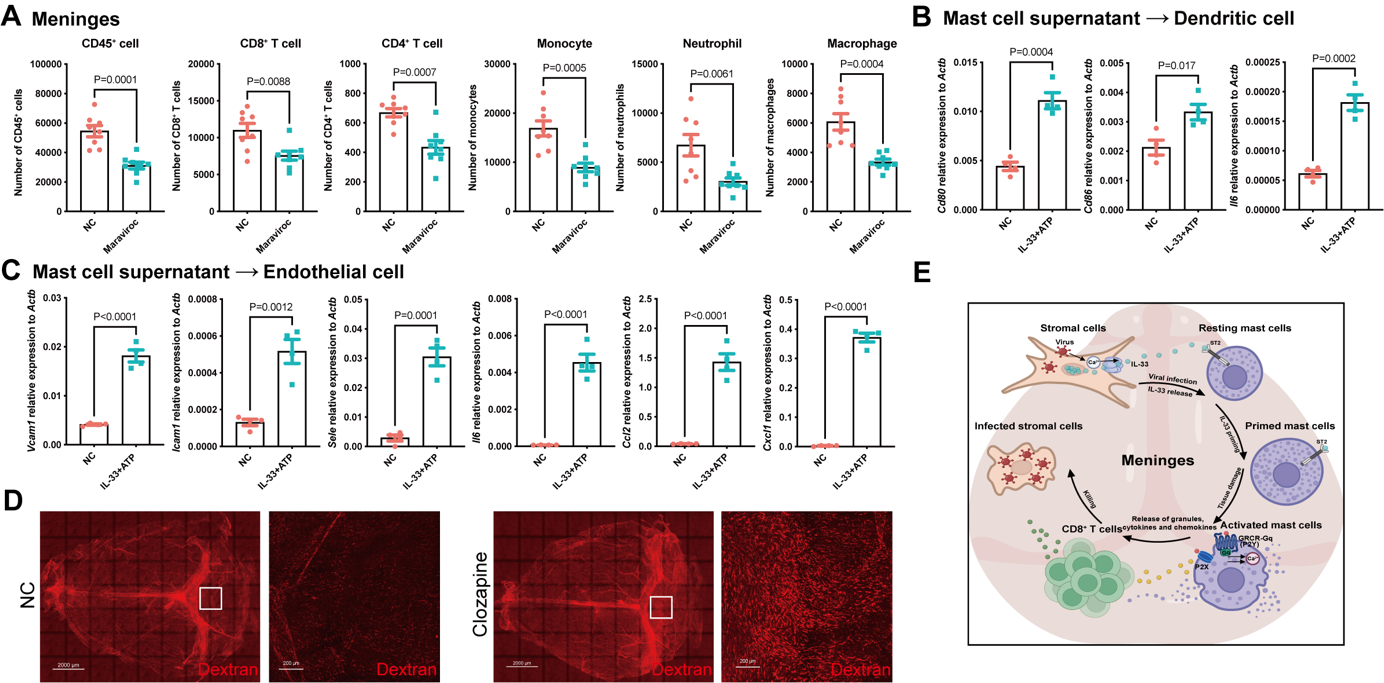
**

A) Meningeal cell counts from treated (CCR5 antagonist, maraviroc) mice at 6 dpi. n=8 (mice). B-C) Supernatant of PCMCs with IL-33 pretreatment and ATP stimulation was used to treat BMDCs and MAECs for RT-qPCR. B) The mRNA levels of *Cd80, Cd86* and *Il6* from BMDCs. n=4 (wells). C) The mRNA levels of *Vcam1, Icam1,* *Sele, Il6, Ccl2* and *Cxcl1* from MAECs. n=4 (wells). D) Maximal intensity projection of dural meninges stained for dextran (red) from clozapine treated *Tpsb2-CreERT2; hM3Dq^fl/+^* mice. Scale bar, 2000 μm (left, whole mount); 200 μm (right). n=2 (mice). E) The model diagram of stromal cell-mast cell communication which orchestrated anti-viral immunity in meninges. Some elements for design are adapted from BioRender.com. Data are presented as mean ± SEM and *p* values were calculated by two-tailed, unpaired Student’s t-test (A-C).

Video 1. Live cell time-lapse imaging of calcium flux (green) in PCMCs from *Tpsb2-CreERT2; Ai162^fl/+^* mice stimulated with conditioned medium from stromal cells. Scale bar, 50 μm.

Video 2. Live cell time-lapse imaging of calcium flux (green) in PCMCs from *Tpsb2-CreERT2; Ai162^fl/+^* mice stimulated with conditioned medium from virus-infected stromal cells. Scale bar, 50 μm.

Video 3. Live cell time-lapse imaging of calcium flux (green) in BMMCs from *Tpsb2-CreERT2; Ai162^fl/+^* mice stimulated with medium. Scale bar, 50 μm.

Video 4. Live cell time-lapse imaging of calcium flux (green) in BMMCs from *Tpsb2-CreERT2; Ai162^fl/+^* mice stimulated with IL-33. Scale bar, 50 μm.

Video 5. Live cell time-lapse imaging of calcium flux (green) in BMMCs from *Tpsb2-CreERT2; Ai162^fl/+^* mice stimulated with ATP. Scale bar, 50 μm.

Video 6. Live cell time-lapse imaging of calcium flux (green) in BMMCs from *Tpsb2-CreERT2; Ai162^fl/+^* mice stimulated with ionomycin and PMA. Scale bar, 50 μm.

Video 7. Live cell time-lapse imaging of calcium flux (green) in the meninges from control mice (*Ai162^fl/+^*) with the stimulation of ATP *ex vivo*. Scale bar, 200 μm.

Video 8. Live cell time-lapse imaging of calcium flux (green) in the meninges from mice expressing Ai162 (*Tpsb2-CreERT2; Ai162^fl/+^*) with the stimulation of 80 μM ATP *ex vivo*. Scale bar, 200 μm.

Video 9. Live cell time-lapse imaging of PCMCs which were not primed for 24 h and then stimulated with medium labeled by WGA (green fire). Scale bar, 5 μm.

Video 10. Live cell time-lapse imaging of PCMCs which were treated with IL-33 for 24 h and then stimulated with medium labeled by WGA (green fire). Scale bar, 5 μm.

Video 11. Live cell time-lapse imaging of PCMCs which were not treated for 24 h and then stimulated with ATP labeled by WGA (green fire). Scale bar, 5 μm.

Video 12. Live cell time-lapse imaging of PCMCs which were treated with IL-33 for 24 h and then stimulated with ATP labeled by WGA (green fire). Scale bar, 5 μm.
